# 6E11, a highly selective inhibitor of Receptor-Interacting Protein Kinase 1, protects cells against cold hypoxia-reoxygenation injury

**DOI:** 10.1038/s41598-017-12788-4

**Published:** 2017-10-10

**Authors:** C. Delehouzé, S. Leverrier-Penna, F. Le Cann, A. Comte, M. Jacquard-Fevai, O. Delalande, N. Desban, B. Baratte, I. Gallais, F. Faurez, M. C. Bonnet, M. Hauteville, P. G. Goekjian, R. Thuillier, F. Favreau, P. Vandenabeele, T. Hauet, M. T. Dimanche-Boitrel, S. Bach

**Affiliations:** 1 Sorbonne Universités, UPMC Univ Paris 06, CNRS USR3151, Protein Phosphorylation and Human Disease Laboratory, Station Biologique, F-29688 Roscoff, France; 2 INSERM UMR 1085, Institut de Recherche sur la Santé, l’Environnement et le Travail, F-35043 Rennes, France; 3 Biosit UMS 3080, Université de Rennes 1, F-35043 Rennes, France; 4 Molecular Signaling and Cell Death Unit, VIB Inflammation Research Center, Ghent, Belgium; 5 Université de Lyon, CNRS UMR 5246, ICBMS, Chimiothèque, Université Claude Bernard Lyon 1, F-69622 Villeurbanne, France; 6 Inserm, U1082 Poitiers, France; 7 CHU de Poitiers, Service de Biochimie, Poitiers, France; 8 Université de Poitiers, Faculté de Médecine et de Pharmacie, Poitiers, France; 9 Fédération Hospitalo-Universitaire SUPORT, Poitiers, France; 10 IBiSA Plateforme ‘MOPICT’, Institut national de la recherche agronomique, Unité expérimentale Génétique, expérimentations et systèmes innovants, Domaine Expérimental du Magneraud, Surgères, France; 11 CNRS UMR 6290, Institut de Génétique et Développement de Rennes, Université de Rennes 1, F-35043 Rennes, France; 12 Division of Infection & Immunity, College of Biomedical and Life Sciences, Cardiff University, Cardiff, United Kingdom; 13 Laboratoire de Biochimie Analytique et Synthèse Bioorganique, Université de Lyon, Université Claude Bernard Lyon 1, F-69622 Villeurbanne, France; 14 Université de Lyon, CNRS UMR 5246, ICBMS, Laboratoire Chimie Organique 2-Glycosciences, Université Claude Bernard Lyon 1, F-69622 Villeurbanne, France; 15 Department of Biomedical Molecular Biology, Ghent University, Ghent, Belgium

## Abstract

Necroptosis is a programmed cell death pathway that has been shown to be of central pathophysiological relevance in multiple disorders (hepatitis, brain and cardiac ischemia, pancreatitis, viral infection and inflammatory diseases). Necroptosis is driven by two serine threonine kinases, RIPK1 (Receptor Interacting Protein Kinase 1) and RIPK3, and a pseudo-kinase MLKL (Mixed Lineage Kinase domain-Like) associated in a multi-protein complex called necrosome. In order to find new inhibitors for use in human therapy, a chemical library containing highly diverse chemical structures was screened using a cell-based assay. The compound 6E11, a natural product derivative, was characterized as a positive hit. Interestingly, this flavanone compound: inhibits necroptosis induced by death receptors ligands TNF-α (Tumor Necrosis Factor) or TRAIL (TNF-Related Apoptosis-Inducing Ligand); is an extremely selective inhibitor, among kinases, of human RIPK1 enzymatic activity with a nM Kd; has a non-ATP competitive mode of action and a novel putative binding site; is weakly cytotoxic towards human primary blood leukocytes or retinal pigment epithelial cells at effective concentrations; protects human aortic endothelial cells (HAEC) from cold hypoxia/reoxygenation injury more effectively than necrostatin-1 (Nec-1) and Nec-1s. Altogether, these data demonstrate that 6E11 is a novel potent small molecular inhibitor of RIPK1-driven necroptosis.

## Introduction

Programmed cell death (PCD) is a natural process for removing unwanted cells in both pathological and non-pathological contexts. Cell death has long been dominated by apoptosis, but include now a growing list of regulated necrosis pathways, including necroptosis, ferroptosis, parthanatos or cyclophilin D (CypD)-dependent necrosis (see^1^ for review). Necroptosis is so far the best-studied form of non-apoptotic cell death. This peculiar PCD does not involve key apoptosis regulators, such as caspases, Bcl-2 family members or cytochrome c release from mitochondria. A series of small chemical inhibitors (termed necrostatins) was used to characterize the Ser/Thr RIPK1 (Receptor-Interacting Protein Kinase 1) kinase as key regulator of necroptosis^2,3^.

Necroptosis is activated upon stimulation of death receptors by the cytokines TNF-α (Tumor Necrosis Factor α), FasL (Fas Ligand) and TRAIL (Tumor-necrosis-factor Related Apoptosis-Inducing Ligand) when caspase-8 is inhibited or absent. The TNFR1 (Tumor Necrosis Factor Receptor 1)-mediated necroptosis is known as the prototype of regulated necrosis. The binding of TNF to its receptor TNFR1 leads to the formation of a sequence of signaling complexes finely tuned by ubiquitylation and deubiquitylation events. The receptor-associated “complex I” induces prosurvival signals through activation of NF-κB (Nuclear Factor – kappa B) and MAPKs (Mitogen Activated Protein Kinases), while other cytosolic complexes drive two different programmed cell deaths: (i) apoptosis, *via* formation of complex including FADD (Fas-Associated Death Domain) that recruits caspase-8 to activate a caspase-dependent cascade; or (ii) necroptosis, via activation of RIPK1 and two other key players, Ser/Thr RIPK3 kinase and the pseudokinase MLKL (Mixed Lineage Kinase Domain-Like) in a complex called the “necrosome”^4^. In accordance with this activation cascade, TNF-α was shown to induce necroptosis in human Jurkat T cells when FADD is deleted^5^.

**Figure 1:**
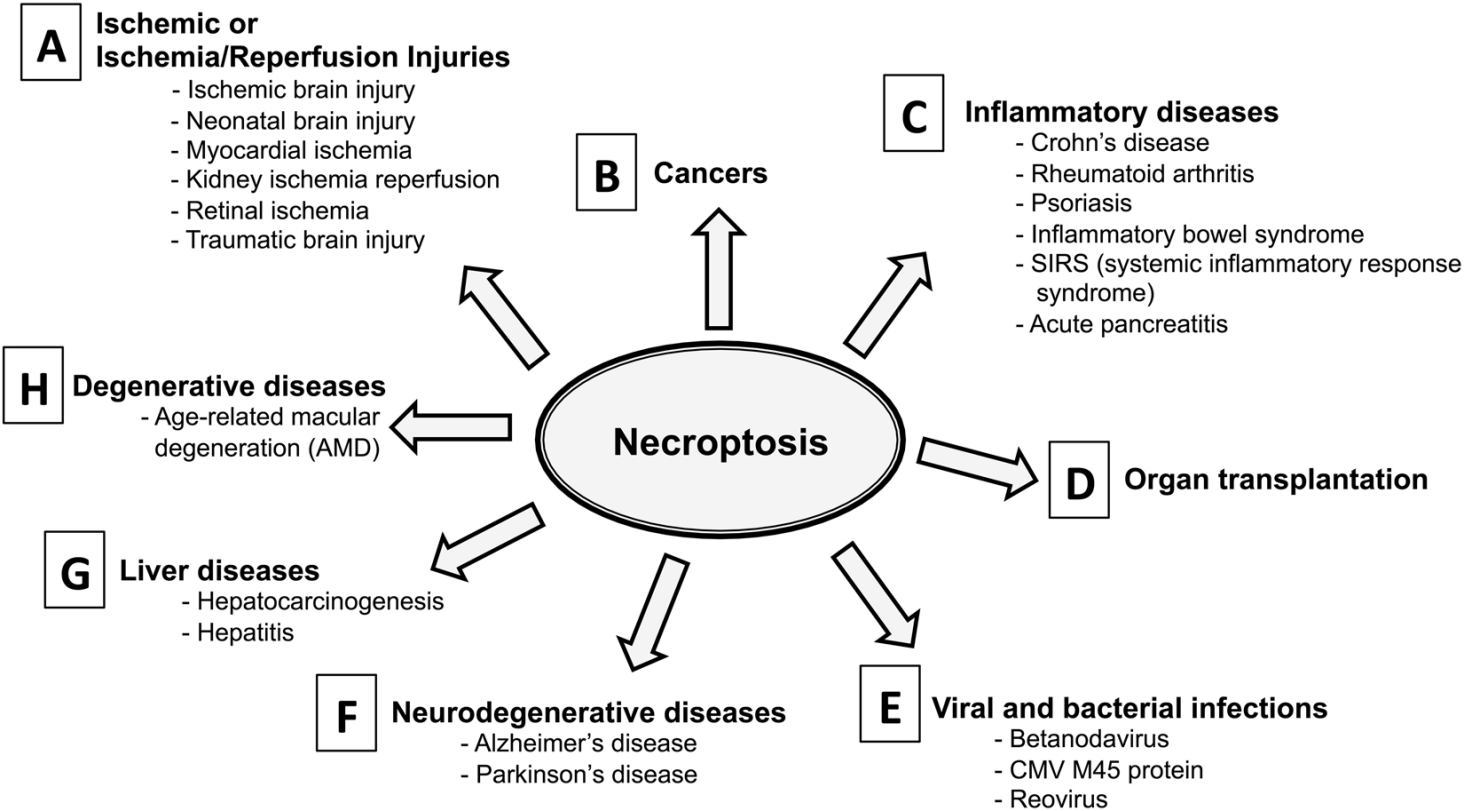
Impact of RIPK1-dependent necroptosis in human diseases. The comprehensive list of the references can be found as Supplementary Table S1.

The ground-breaking finding that necroptosis is a genetically controlled process led to the hypothesis that this programmed cell-death is ‘druggable’, an emerging breakthrough that carries the potential for significant advances in everyday clinical medicine^2^. Indeed molecular targets, including RIPK1, RIPK3 and MLKL, have been shown to be involved in multiple disease models where necroptosis is of central pathophysiological relevance, such as: in ischemia-reperfusion injury (including stroke, myocardial infarction, resuscitation, solid organ transplantation or heart surgery) in brain, heart and kidney diseases, and in inflammatory diseases, including moderate to severe Rheumatoid Arthritis (RA), psoriasis, retinal disorders, neurodegenerative diseases and infectious disorders (sepsis, viral infections, parasites, bacterial infections)^6,7^ (Fig. 1). More recently, it has been shown that human and murine tumor cells induce necroptosis of endothelial cells, which promotes tumor cell extravasation and metastasis^8^. Necroptosis can thus also be targeted in the treatment of human metastasis, the leading cause of cancer-related death in humans.

Following the molecular description of necroptosis and the characterization of necrostatins^2,3,9^, various screening initiatives using cell-based assays or high-throughput *in vitro* RIPK1 binding assays have shed light on new chemical scaffolds including the 1-aminoisoquinolines^10^, 5-phenylpyrrolo[2,3-b]pyridines^10^, 5-arylpyrrolo[2,3-b]pyridines^10^, furo[2,3-d]pyrimidines^10^, analogs of Bcr-Abl inhibitor ponatinib^11,12^, PN10 (hybrid of ponatinib and Nec-1s)^11^, the benzo[b][1,4]oxazepin-4-ones^13^, the compound GSK’963^14^ and the RIPA-56^15^. The quest for an optimized clinical candidate is still in progress with the synthesis of the first-in-class RIPK1 specific inhibitor (GSK2982772)^16^.

We now reported here the selection of a new highly selective inhibitor of RIPK1, 2-(4-(benzyloxy)phenyl)-2,5-dihydroxy-7-methoxychroman-4-one (compound called 6E11), using a phenotypic cell screen which detects the ability of small chemicals to block TNF-driven necrotic death. Moreover, we show that this new inhibitor protects cells from cold hypoxia/reoxygenation injury. This work sheds light on the interest to study natural products or derivatives in the quest of drugs targeting necroptosis-related disorders.

## Results

### Discovery of a novel potent small molecular inhibitor of necroptosis

**Figure 2:**
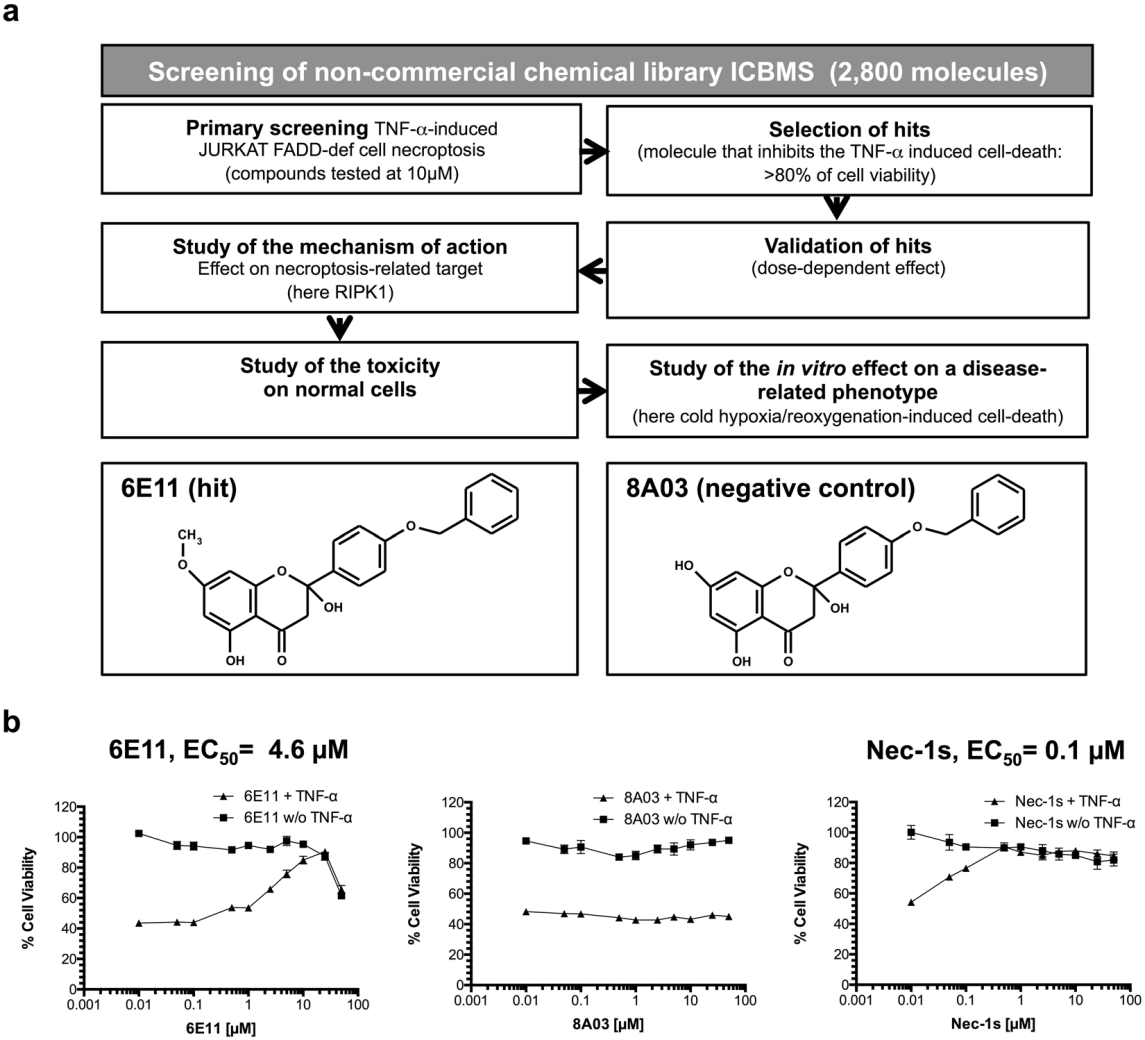
Characterization of hit compound 6E11 as new necroptosis inhibitor. (**a**) Workflow of the cell-based screening of ICBMS chemical library for the selection of new inhibitors of necroptosis. Among 2,800 compounds, 6E11, was selected as the more potent inhibitor of TNF-α-induced necroptosis in human FADD-deficient Jurkat T cells. The chemical structures of 6E11 and its negative control (8A03) are depicted above the workflow. The primary screening is performed in monoplicate. The negative control was not detected during the screening campaign. (**b**) Dose-dependent protection of 6E11 against TNF-α-induced Jurkat FADD deficient cell necroptosis. After a 24-h incubation of the cells with or without (w/o) TNF-α and increasing concentrations of tested compounds, the effect on the cell viability was evaluated by MTS reduction assay. The cells were treated only with the tested compound to evaluate its putative toxicity. The values were normalized as a percentage of cell viability, considering 100% viable cells in the control treated with DMSO (n = 3, mean ± SD).

To identify new necroptosis inhibitors, a robust TNF-induced FADD-deficient human Jurkat necroptosis assay was used to screen a chemical library of 2,800 compounds belonging to the chemical library of University Claude Bernard (ICBMS, Lyon, France) (Fig. 2a). The screening process was assessed using a viability test based on cell proliferation measurement (MTS). Among the tested molecules, compound 6E11 was found to be the more potent to block the TNF-induced necrotic cell death with an EC_50_ of 4.6 μM. Although less potent than Nec-1s (EC_50_ of 0.1 μM), 6E11 is a novel core for structural optimization (Fig. 2b). Its inactive analogue 8A03 did not show any inhibition towards necrotic cell death. The chemical structures of these compounds are depicted on Fig. 2a. 6E11 is a natural product derivative as it’s a synthetic derivative of the naturally occuring 2,5-dihydroxy-2-phenylchroman-4-ones isolated from *Populus nigra* buds^17^. The bioactivity of 6E11 was studied in the work reported here.

### 6E11 is selective towards death receptor-induced necroptosis

**Figure 3:**
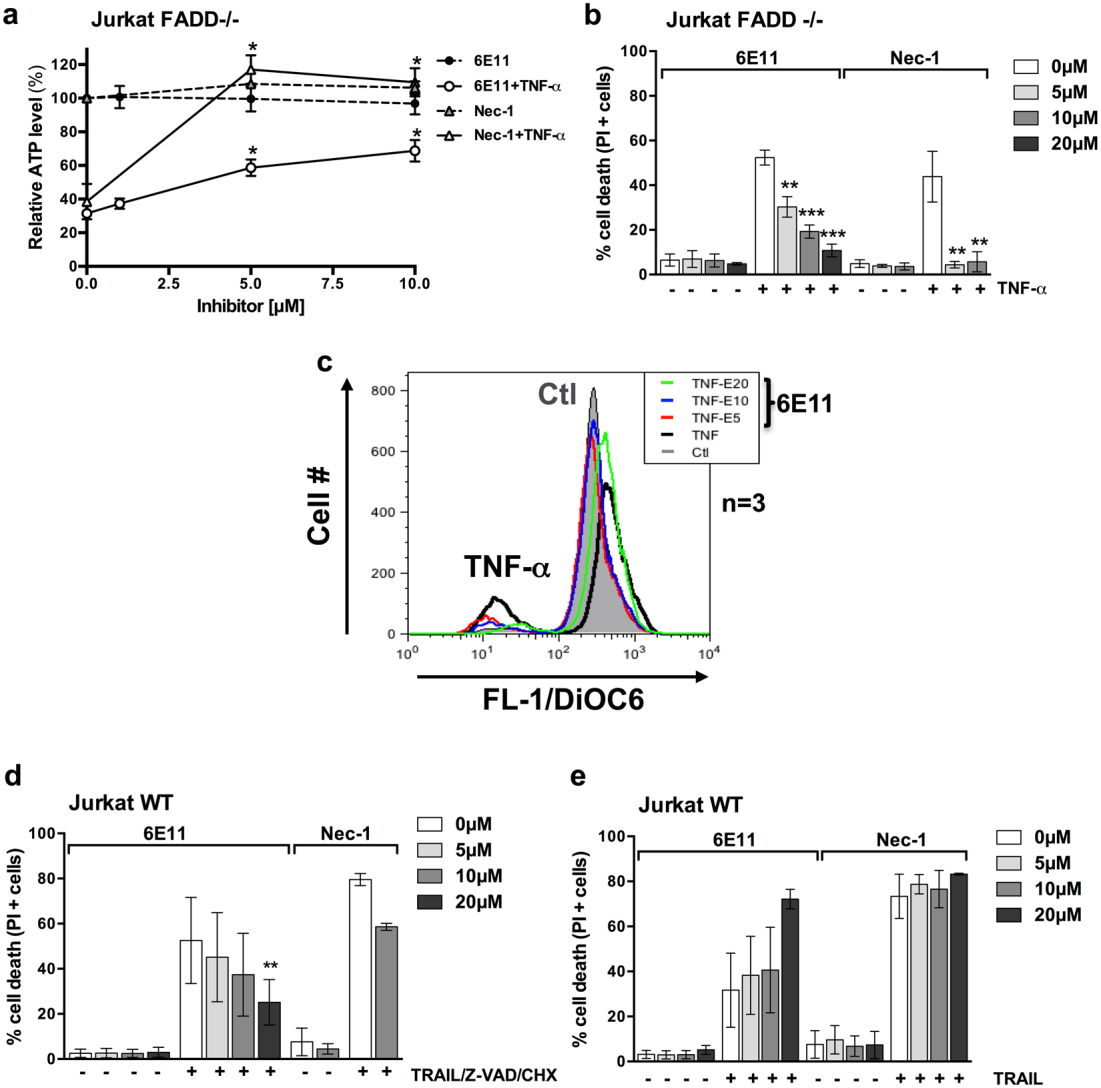
6E11 inhibits death receptor-induced necroptosis, but not apoptosis. (**a**) Human FADD-deficient Jurkat T cells were treated or not with TNF-α (10 ng/ml) in presence or not of increasing concentrations of 6E11 (0, 1, 5, 10 µM) or Nec-1 (0, 5, 10 µM) for 18 hours. Intracellular ATP levels were measured with the CellTiter-Glo® Luminescent Cell Viability Assay (n = 3, mean ± SEM, *P < 0.05) (EC_50_ ~6 µM). (**b**) Human FADD-deficient Jurkat T cells were treated or not with TNF-α (10 ng/ml) in presence or not of increasing concentrations of 6E11 (0, 5, 10, 20 µM) or Nec-1 (0, 5, 10 µM) for 18 hours. The percentage of cell death was determined by propidium iodide staining using flow cytometry (n = 3, mean ± SEM, **P < 0.01 and ***P < 0.001). (**c**) Human FADD-deficient Jurkat T cells were treated or not with TNF-α (10 ng/ml) in presence or not of increasing concentrations of 6E11 (0, 5, 10, 20 µM) for 18 hours. The mitochondrial transmembrane potential (MTP) was measured using the fluorescent dye DiOC6^(3)^ and flow cytometry analysis. (**d**) 6E11 inhibits TRAIL-induced necroptosis. Wild-type Jurkat T cells were treated or not with TRAIL (10 ng/ml), Z-VAD (30 µM) and CHX (1 µg/ml) for 18 hours in presence or not of increasing concentrations of 6E11 (0, 5, 10, 20 µM) or 10 µM Nec-1. The percentage of cell death was determined by propidium iodide staining using flow cytometry (n = 3, mean ± SEM, **P < 0.01). (**e**) 6E11 does not inhibit TRAIL-induced apoptosis. Wild-type Jurkat T cells were treated or not with TRAIL (20 ng/ml) for 18 hours in presence or not of increasing concentrations of 6E11 (0, 5, 10, 20 µM) or Nec-1 (0, 5, 10, 20 µM). The percentage of cell death was determined by propidium iodide staining using flow cytometry (n = 3, mean ± SEM).

6E11’s ability to inhibit necroptosis was compared to Nec-1 in human cell lines *in vitro*. This antinecroptotic activity of 6E11 was demonstrated by measurements of intracellular adenosine triphosphate (ATP) levels (Fig. 3a), plasma membrane permeabilization (% of propidium iodide positive cells) (Fig. 3b) and mitochondrial transmembrane potential in living cells (DiOC6(3) measurement) (Fig. 3c). Necroptosis was induced by necroptotic triggers such as TNF-α in human FADD-deficient Jurkat T cells resistant to extrinsic apoptosis (Fig. 3a–c) or TRAIL + Z-VAD + CHX in human WT Jurkat T cells (Fig. 3d). In both models, 6E11 blocked necroptosis in a dose-dependent manner (Fig. 3a–d). Moreover, as Nec-1, 6E11 did not rescue WT Jurkat cells from apoptosis induced by TRAIL (Fig. 3e). We shown also that 6E11 did not protect murine cell lines (L929 or MEFs) from necroptotic stimulus (see Supplementary Fig. S1 describing the data obtained on L929 cells). These results indicate that 6E11 blocks necroptotic but not apoptotic cell death and suggest that this anti-necroptotic efficacy is species-specific.

### 6E11 is not cytotoxic at inhibitory concentrations and rescues cells from necroptosis even after necrotic stimulus induction

**Figure 4:**
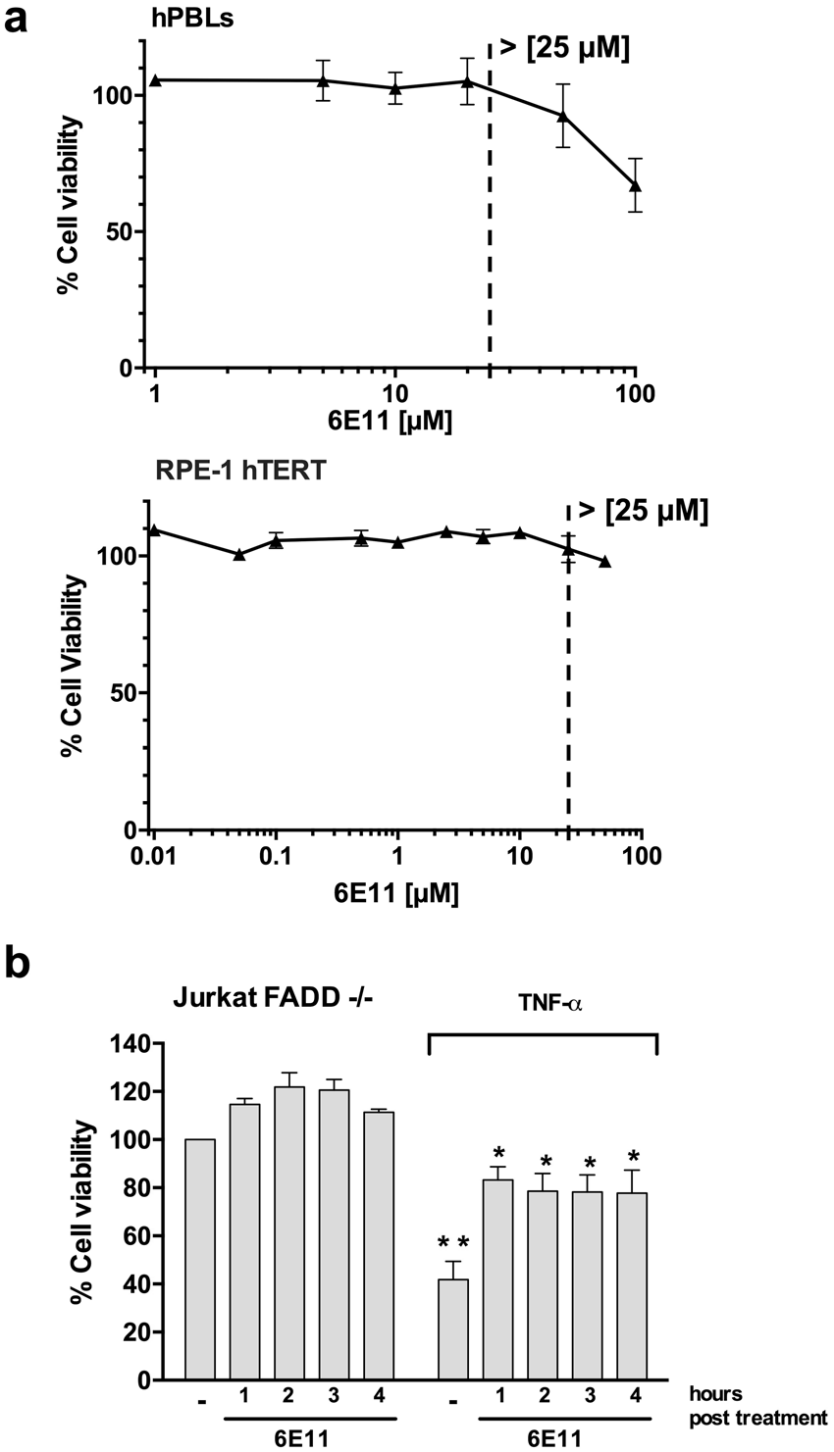
6E11 is not cytotoxic at inhibitory concentration and protects from necroptosis even after cell death initiation. (**a**) 24 hours after treatment with increasing concentrations of 6E11, cell viability was measured with a MTS assay to determine the toxicity of the compound towards either human PBL (upper panel) (n = 6 individuals, mean ± SEM) or human retina pigmented epithelial cells (RPE-1 hTERT) (lower panel) (n = 3, mean ± SD). (**b**) Viability was measured with a MTS assay in FADD-def Jurkat cells treated with TNF-α (10 ng/ml) followed by 10 µM 6E11 addition 1 to 4 h post necroptosis initiation (n = 4, mean ± SEM, *P < 0.05; **P < 0.01).

6E11 has a little or no cytotoxic effect in the range of 1 to 25 µM against human peripheral blood leukocytes (PBL) or human retinal pigment epithelial cells (RPE-1 hTERT) (Fig. 4a), concentrations at which it significantly suppresses necroptosis. In order to investigate 6E11 activity as a necroptosis inhibitor in a potentially relevant clinical scenario, we investigated its efficacy after, rather than before, necroptosis induction. 6E11 was added at a concentration of 10 µM from 1 h to 4 h after TNF treatment in FADD-deficient Jurkat cells. When added from one to four hours following necroptosis induction, 6E11 rescues FADD-deficient Jurkat cells from 60% cell death to approximately 20% (Fig. 4b).

### 6E11 is a highly selective inhibitor of human RIPK1 among protein kinases

**Figure 5:**
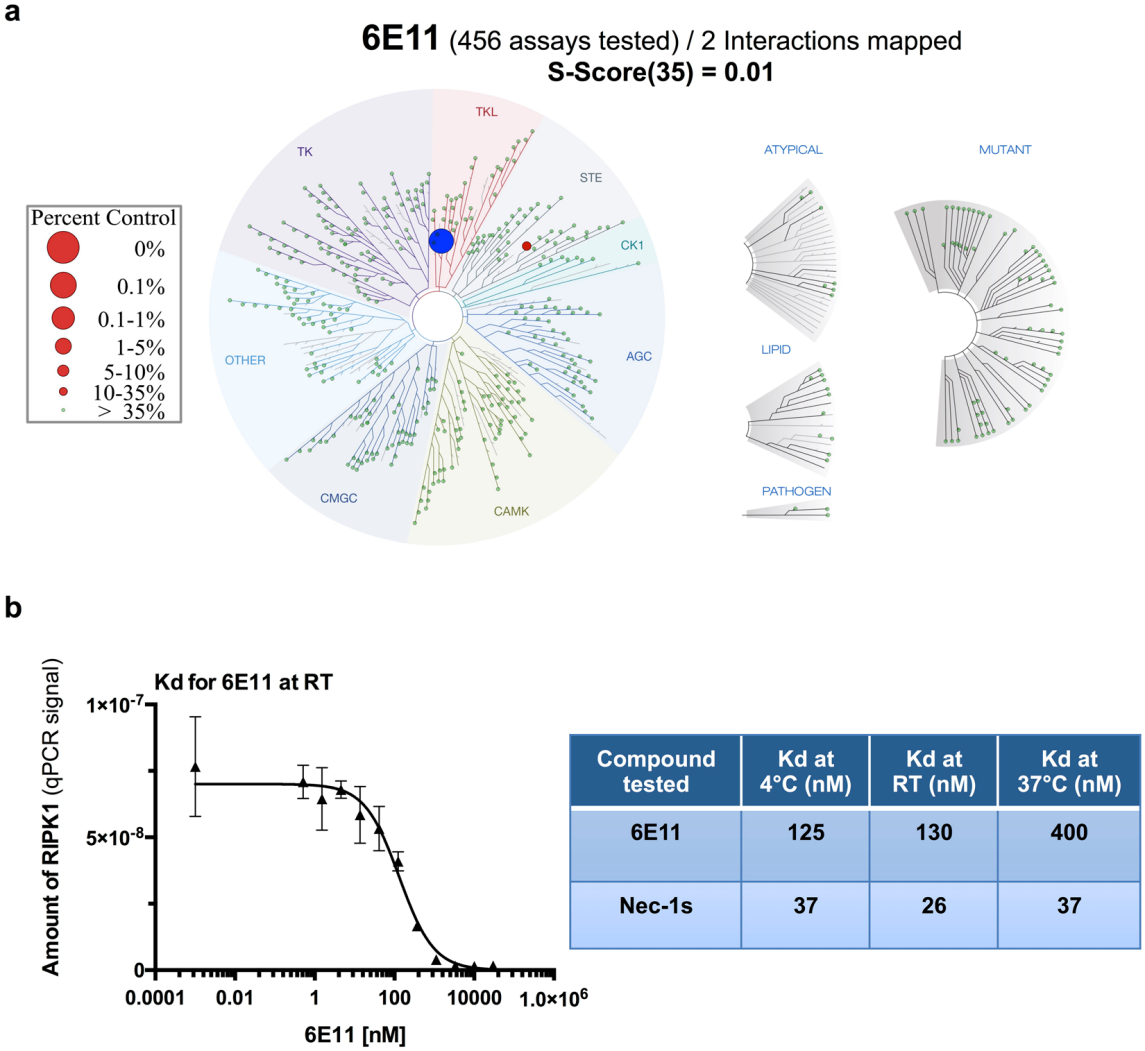
6E11 is a highly selective inhibitor of human kinase RIPK1. (**a**) Selectivity of 6E11 was assessed biochemically against a panel of 456 purified kinases (KINOMEscan^SM^ Assay) at 10 µM and the compound was found to be highly selective for RIPK1. Only 0.15% of the initial amount of RIPK1 is still on the affinity matrix after competition with 6E11 (the full list of results is reported in Supplementary Table S2). S-Score^35^ = (number of non-mutant kinases with %Ctrl < 35)/(number of non-mutant kinases tested). (**b**) Measure of the binding constant for RIPK1/6E11 and RIPK1/Nec-1s interactions. Two 11-point 3-fold serial dilutions of 6E11 and Nec-1s were prepared in 100% DMSO in order to determine the binding constant (Kd). Kds are calculated at three various temperatures by measuring the amount of kinase captured on the solid support as a function of the test compound concentration (n = 2, mean ± range).

To elucidate the binding mode of 6E11 and to identify its target, the selectivity of 6E11 was assessed at 10 µM against a panel of 456 kinases (mainly human) in an *in vitro* competition binding assay (KINOMEscan®, DiscoverX, San Diego, CA, USA). 6E11 showed an almost absolute kinase selectivity towards human RIPK1. Only Mek5 was shown to be poorly affected by 6E11 (see Supplementary Table S2 for details). In these results, 6E11 competes with 99.85% of RIPK1-ligand complexes leading to prevention of kinase binding to the immobilized ligand (Supplementary Table S2 and blue circle on Fig. 5a). The dissociation constant (Kd) of 6E11 for RIPK1 was calculated with a standard dose-response curve using this competition-binding assay. 6E11 shows a high affinity for RIPK1 with a Kd of 130 nM (Fig. 5b, left and right panels). High affinity Nec-1s for RIPK1 was also confirmed with Kd equal to 26 nM (at room temperature). Contrarily to Nec-1s, the binding affinity of 6E11 to RIPK1 is sensitive to the temperature. The affinity of 6E11 for RIPK1 is higher at 4 °C and room temperature than at 37 °C (Fig. 5b, right panel).

**Figure 6:**
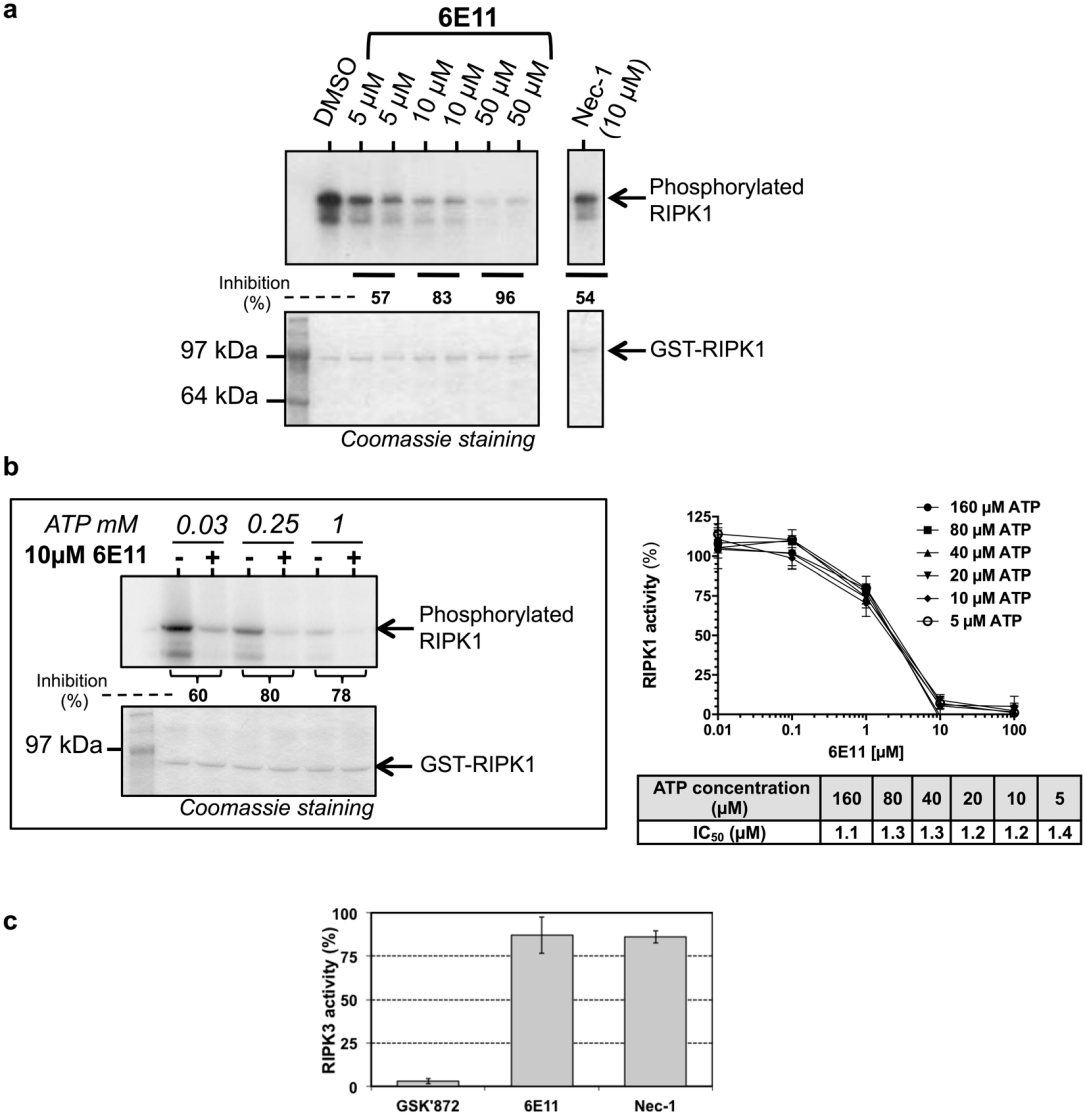
6E11 inhibits the enzymatic activity of RIPK1 with a non-ATP competitive mode of action. (**a**) RIPK1 was treated with 5, 10 or 50 µM of 6E11 to analyze the effect on the kinase autophosphorylation. Radioactive autophosphorylation assays were processed with [γ-^32^P] ATP at 30 µM final concentration. Necrostatin-1 (Nec-1) was used as an internal control. Coomassie blue staining was performed in order to estimate the total amount of protein loaded on polyacrylamide gel. Autophosphorylated RIPK1 band was visualized on radiographic film. % of RIPK1 activity inhibition are calculated through a ratio with DMSO, as a 0 percent reference. See Supplementary Fig. S2 for results of the full-length gel obtained at various exposure times. (**b**) ATP competition assay shows that inhibition of RIPK1 activity by 6E11 is not affected by ATP concentration. On the left panel, 0.25 or 1 mM of cold ATP were used to study the competition with 6E11. These assays are similar to those described in (**a**). The phosphorylation signal is modified by increasing doses of ATP due to the dilution of [γ-^32^P] ATP by “cold” non radiolabeled ATP. % of RIPK1 activity inhibition are calculated for each ATP concentration through a ratio with DMSO (**-**), as a 0 percent reference. See Supplementary Fig. S2 for results of the full-length gel. The RIPK1 catalytic activity was also monitored using myelin basic protein, MBP, as a substrate (right panel). GST-RIPK1 full length produced by baculovirus in Sf9 insect cells was assayed in the presence of increasing concentrations of 6E11. The kinase activities are expressed in % of maximal activity, i.e. measured in the absence of inhibitor. The IC_50_ values obtained at various ATP concentrations are reported in the table (n = 3, mean ± SD). (**c**) 6E11 is inactive on RIPK3 activity. GST-RIPK3 full length produced by baculovirus in Sf9 insect cells was assayed in the presence of 10 µM of inhibitor with [γ-^33^P] ATP and myelin basic protein, MBP, as a substrate. GSK’872 was used as RIPK3 reference inhibitor. Kinase activities are expressed in % of maximal activity, *i*.*e*. measured in the presence of DMSO (as a 100% reference).

To monitor RIPK1 enzymatic activity in presence of 6E11, we performed an *in vitro* auto-phosphorylation assay using recombinant full-length RIPK1 (GST-tagged). This assay showed an inhibition of RIPK1 auto-phosphorylation by 6E11 in a dose-dependent manner (Fig. 6a). The enzymatic activity of RIPK1 was also quantified by monitoring the phosphorylation of Myelin-Basic Protein (MBP), a known substrate of RIPK1. Accordingly, 6E11 efficiently inhibited the phosphorylation of MBP in a dose-dependent manner with an IC_50_ equal to 1.2 µM with 20 µM ATP (Fig. 6b). To further characterize the mechanism of RIPK1 inhibition by 6E11, we monitored RIPK1 activity in the presence of 6E11, with increasing concentrations of cold ATP (up to 1 mM). Results obtained show that, in the presence of increasing concentrations of ATP, the inhibitory activity of 6E11 on RIPK1 auto-phosphorylation was maintained. 6E11 is thus a non-ATP competitor (Fig. 6b). Nec-1 was described as an ATP competitive compound^3^. Very interestingly, 6E11 had no significant effect at 10 µM on RIPK3 kinase activity (Fig. 6c). Taken altogether, these data demonstrated that 6E11 is a specific inhibitor of RIPK1 kinase activity acting through a different inhibition mechanism than Nec-1.

### 6E11 has a putative binding site out of the ATP binding cleft of RIPK1

**Figure 7:**
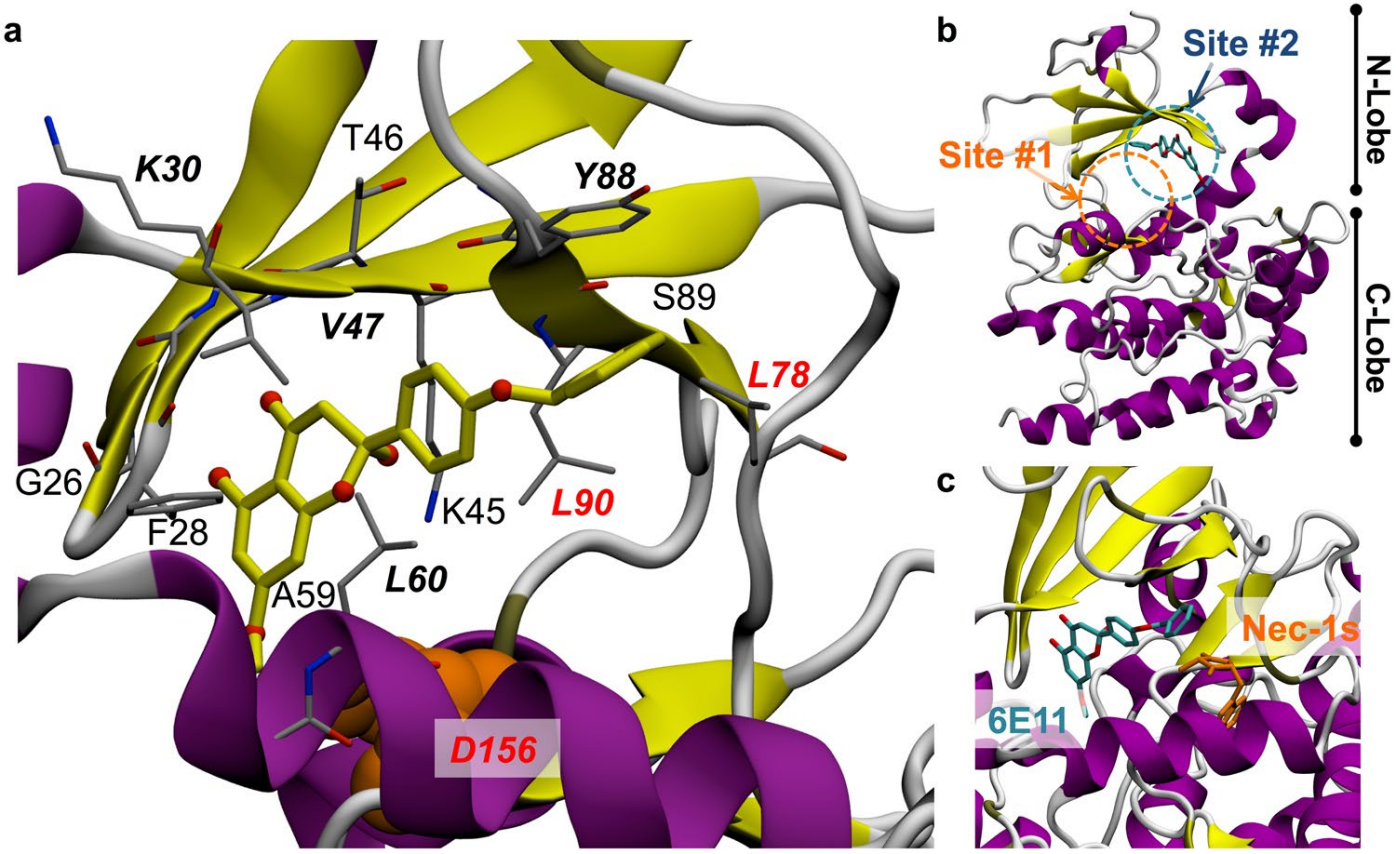
Molecular model of the RIPK1-6E11 complex. (**a**) Close-up view of the putative interaction between 6E11 and surrounding residues in RIPK1 extracted from the best pose of docking simulation improved by 60 ns molecular dynamic trajectory (key-residues are shown in italic and bold, residues common to Nec-1s and 6E11 binding sites are in red). Asp156, one of the RIPK1 catalytic triad residues, but more slightly contacting 6E11, is shown in orange spheres to complete the description of the close environment of the ligand. (**b**) Overall structure of RIPK1 showing the putative binding site of 6E11 highlighted in blue dashed circle (site #2). The binding site (site #1) of Nec-1s (determined from the X-ray structure 4ITH) is marked with a orange dashed circle on the same panel. (**c**) Relative positions and orientations of each 6E11 and Nec-1s compounds shown in a close-up view.

The predictive orientation for the hit compound described here was studied by *in silico* analysis of the theoretical RIPK1-6E11 complex. Stable contact residues defining a pharmacophore and determined on the most representative structural model included six key amino acids Lys30, Val47, Leu60, Leu78, Tyr88 and Leu90 (Fig. 7a). Surrounding residues (4.0 Å cut-off distance) describing the 6E11 binding pocket observed over the molecular docking simulation trajectory also comprised of Phe28, Val31, Lys45, Thr46, Ala59, Glu63, Val81, Ile83, Ser89 and Asp156. We should note that among these amino acids, three of them (Leu78, Leu90 and Asp156) have been already described to be involved in the interaction with necrostatins (see^18^ for details on RIPK1-Necrostatins complexes) (Fig. 7a). Molecular dynamic (MD) simulation of RIPK1-6E11 model allowed us to improve the preferential binding mode identified by docking calculations which is different from Nec-1s (Fig. 7b,c). The binding site #1 of Nec-1s is marked with a orange circle on Fig. 7b. From analysis of the most frequent contacts of the compound to the kinase, we are able to propose that 6E11 should bind RIPK1 kinase through tight hydrophobic interactions and a non-specific hydrogen bond (HB), as well as other transient HB interactions observed during the simulation (Fig. 7b,c). The putative binding site #2 of 6E11 is marked with a blue circle on Fig. 7b. Our simulations suggest that 6E11 fits tightly in an alternative and putative cleft surrounded notably by the RIPK1 catalytic triad residues: Lys45, Glu63 and Asp156. This cleft of RIPK1 is mainly hydrophobic but richer in hydrogen bond acceptors than the kinase hinge within the ATP-binding site. Interestingly, this model shows that 6E11 does not make any interaction with the kinase hinge in this conformation of RIPK1 regardless of hydrogen bonds. Moreover, this proposed binding mode for 6E11 occupying a lipophilic pocket in a cleft near the substrate binding site of RIPK1 indicates that this compound is likely a type III kinase inhibitor. This binding mode is in line with the high selectivity of 6E11 detected by the KINOMEscan^SM^ Assay (Fig. 5a) and also with the non-ATP competitive mode of inhibition (reported on Fig. 6b).

### 6E11 protects human aortic endothelial cells (HAEC) from cold hypoxia-reoxygenation

**Figure 8:**
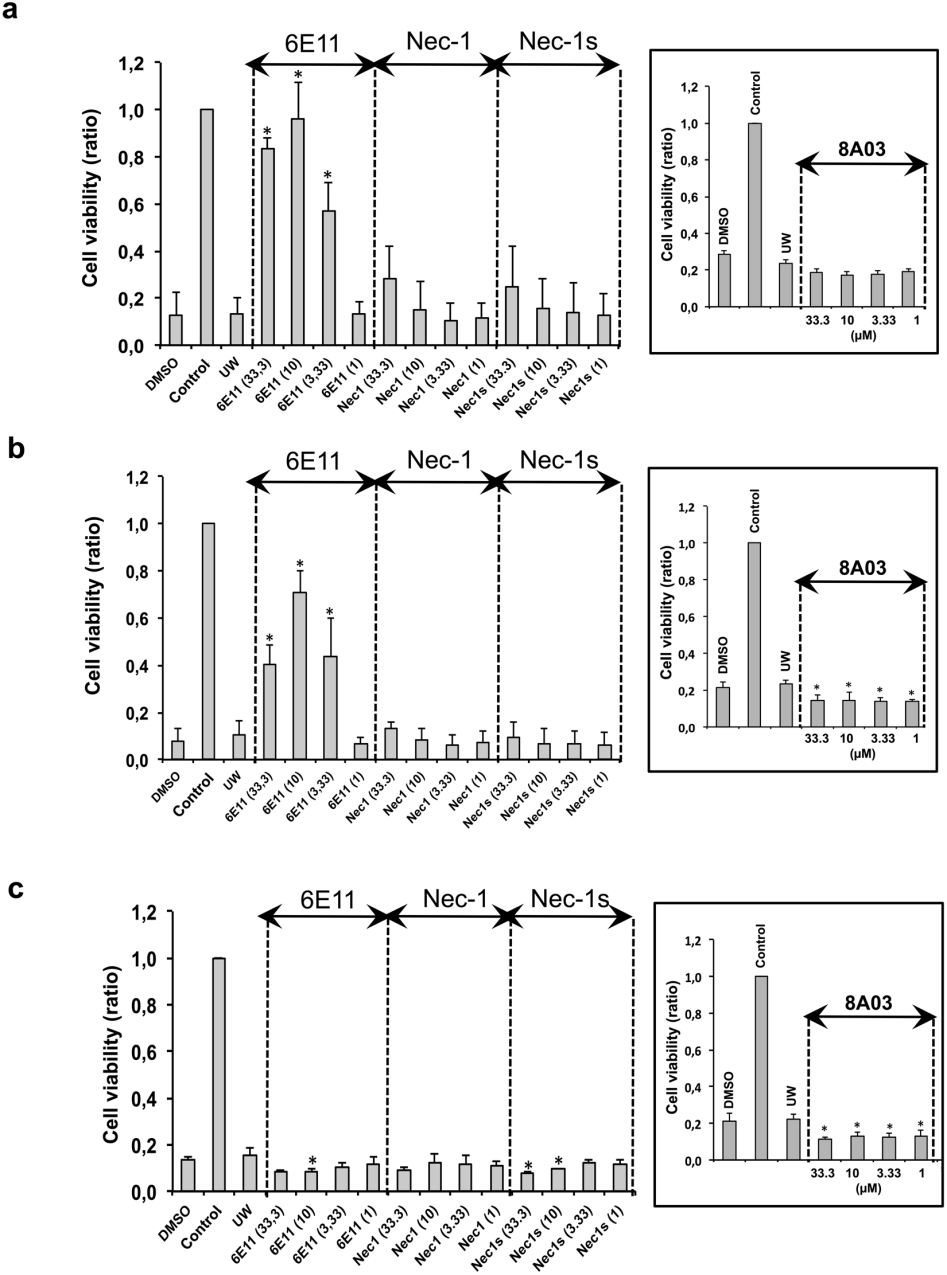
6E11 protects HAEC from cold hypoxia reoxygenation injury when treatment occurs during cold hypoxia step (**a**), during both cold hypoxia and reoxygenation steps (**b**), but not when treatment occurs during the reoxygenation step (**c**). Increasing concentrations (1, 3.33, 10, 33.3 µM) of 6E11, Nec-1, Nec-1s or 8A03 were added to the UW preservation solution during hypoxia or/and in PBS – 2% FBS during reoxygenation. UW corresponds to cells treated with UW preservation solution. Controls are cells not subjected to this protocol and continuously oxygenated. DMSO are cells treated with drug vehicle. The ratio of cell viability evaluated by XTT test, is calculated in comparison to the untreated control (100% of cell viability). Data shown are mean ± SD, n = 3; ANOVA and Tukey’s Multiple Comparison Test; *P < 0.05 vs UW. See Supplementary Fig. S3 for a schematic representation of the protocol.

Hypoxic cold storage condition mimics the situation occurring during *ex vivo* graft preservation and the cold hypoxia/reoxygenation sequence mimics organ transplantation process, two situations in which necrotic cell death occurs. HAEC cells were washed in PBS then incubated with University of Wisconsin (UW) preservation solution in anoxic and hypoxic conditions for 24 h (simulating cold ischemia organ preservation), after which the medium was changed and cells put in regular culture conditions (simulating reperfusion). To assess the potential protective activity of our compounds against cold ischemia, increasing concentrations of 6E11, Nec-1, Nec-1s, or 8A03 were added to the UW for the preservation steps. The compounds were added at various steps of the hypoxia reoxygenation sequence (see Supplementary Fig. S3 for a detailed view of the experiment). The treatment with compound 6E11 during cold hypoxia or during cold hypoxia and reoxygenation brought measurable benefits on cell survival. Compared to the control inhibitors of necroptosis (Nec-1 and Nec-1s), the effect of compound 6E11 was significantly better (Fig. 8a and b). No protective effect was observed when 6E11 was added only at the reoxygenation step (Fig. 8c). The structurally related compound 8A03 was shown to be inactive.

## Discussion

Nearly 50% of all approved drugs from the 1940s to 2014 are of natural origin^19^, actually being either natural products or directly derived therefrom. This percentage highlights the crucial impact of studies of natural products on the discovery of new drugs. In line with this observation, the present study reports the characterization of a novel highly selective natural product derivative inhibitor of RIPK1. By using a cell-based screening assay to analyze a French academic chemical collection of 2800 small molecular compounds, we identified 6E11 as a new inhibitor of programmed necrosis with a micromolar EC_50_ concentration. 6E11, 2-(4-(benzyloxy)phenyl)-2,5-dihydroxy-7-methoxychroman-4-one, is a compound derived from the 2,5-dihydroxy-2-phenylchroman-4-ones isolated from *Populus nigra* buds^17^. This study enlarges the chemical families of potent necroptosis inhibitors. 6E11 was shown to inhibit TNF-α or TRAIL-induced necroptosis in human Jurkat cells but not TRAIL-induced apoptosis. In both models, 6E11 inhibited hallmarks of necroptosis in a dose-dependent manner, notably the plasma membrane permeabilization and the decrease of intracellular ATP levels. In order to study the mechanism of action of 6E11, a KINOMEscan® was performed and showed that RIPK1 was specifically affected by the selected chemical compound with a low nanomolar Kd (130 nM at room temperature). Mek5 was the only other kinase weakly affected among the large set tested (456 kinases, wild type and mutants). In line with this result, we showed that RIPK3 kinase activity is not inhibited by 6E11 and that the inhibition of RIPK1 is not affected by high concentrations of ATP (up to 1 mM). Based on its high selectivity, on the non-ATP competitive mechanism of inhibition, we hypothesized that 6E11 targets a pocket outside the ATP-binding cleft. The high sequence similarity in the ATP-binding site among members of the kinase families, a pocket targeted by the vast majority of kinase inhibitors discovered to date, often results in low selectivity and additional toxicities, and suffer from competition with high intracellular ATP concentrations. In order to predict a putative binding mode of 6E11 to the RIPK1 kinase domain we performed molecular simulations. The result obtained is shown on Fig. 7. In this model, 6E11 occupies a lipophilic pocket in the “back” cleft of RIPK1 and thus can be classified as type III kinase inhibitor (referred to as allosteric inhibitors) in a distinct site from Nec-1s. It interacts putatively with the three catalytic amino acids, Lys45, Glu63 and Asp156, in proximity to the triphosphate and substrate binding site. In contrast to Nec-1s, 6E11 does not compete with intracellular ATP (typically in the low-millimolar range) to negatively modulate RIPK1 activity. Interestingly, Degterev and Linkermann^20^ report *in vivo* studies showing that selective inhibition of RIPK1 should not be considered as the sole parameter in tackling deleterious effects of renal ischemia–reperfusion injury: Nec-1 is active whereas the more RIPK1-selective Nec-1s is not. In this study, we show that 6E11 has a stronger protective effect against cell death induced by cold hypoxia/ reoxygenation in HAEC than both Nec-1 and Nec-1s (Fig. 8). Ren *et al*.^15^ used a cell-based screen on a collection of 200,000 compounds in order to identify another chemical scaffold of type III inhibitors, RIPA-56 (N-benzyl-N-hydroxy-2,2-dimethylbutanamide), exhibiting an absolute selectivity for RIPK1. Harris *et al*.^13^ have characterized Benzo[b][1,4]-oxazepin-4-ones as type III mono-kinase selective RIPK1 inhibitors from a GSK’s property collection of DNA-encoded small-molecule libraries. Similarly to this class of RIPK1 inhibitors, we showed that 6E11 is also specific of human kinase. It will be very informative to test other type III inhibitors in the cold hypoxia/ reoxygenation assay described here (see Supplementary Fig. S3). The concept of necroinflammation has recently emerged in ischemia/reperfusion injury. Necroinflammation can be initiated by a few necrotic cells that activate the innate immune system, which subsequently leads to necrosis of more cells triggering more inflammation in a process leading to organ failure. In this context, pharmacologic inhibition of regulated necrosis, if directly associated with inflammation, might functionally be considered as an anti-inflammatory treatment.

There are great clinical and basic research needs to characterize selective, high-affinity, small-molecule inhibitors of programmed necrosis-related kinases, including RIPK1. 6E11 is one of such compounds useful for pathway elucidation and target validation in addition to drug discovery. The RIPK1 inhibitor with this newly identified scaffold needs to be chemically optimized in order to study its potential use in clinic for improving the solid organ preservation and transplantation in conditions of static or dynamic cold ischemia. Our main objective is to increase the use of organs previously considered as marginal (e.g. from expanded criteria donors) in the context of the current organ shortage crisis. As mentioned on Fig. 1, necroptosis is implicated in a large spectrum of human disorders. Thus the bioactivity of such drug-like compound could also be investigated in other clinical use, such as psoriasis and ulcerative colitis.

## Methods

### Cell cultures

Jurkat *wild-type* A3, FADD-deficient Jurkat I 2.1 and RPE-1 hTERT human cell lines were obtained from ATCC (American Type Culture Collection, Rockville, MD, USA). Jurkat *wild type*, FADD-deficient Jurkat cells were cultured in RPMI containing Glutamax (Invitrogen) supplemented with 15% FCS. RPE-1 hTERT cells were cultured in DMEM-F12 containing Glutamax (Invitrogen) supplemented with 10% FCS. Peripheral blood mononuclear cells (PBMCs) were isolated by Ficoll gradient centrifugation from blood buffy coats of healthy donors, provided by the Etablissement Français du Sang (EFS). The research protocol was conducted under French legal guidelines. After separation of monocytes by 1 h adhesion step, non-adherent PBMCs (PBL) were harvested. PBL were cultured in RPMI 1640 medium (Gibco, Life technologies, Carlsbad, CA, USA) supplemented with 10% decomplemented fetal calf serum (Life technologies), penicillin (100 IU/ml), and streptomycin (100 µg/ml). All cells were cultured under a 5% CO_2_ atmosphere at 37 °C.

### Reagents

Recombinant human Flag-tagged TRAIL (TRAIL-Flag) and z-VAD-fmk were obtained from Enzo Life Sciences (Villeurbanne, FR). Necrostatin-1 (Nec-1) and necrostatin-1s (Nec-1s) were from Calbiochem (VWR International, Fontenay-sous-Bois, FR). Anti-Flag M2 IgG1 antibody, cycloheximide (CHX), propidium iodide, were obtained from Sigma-Aldrich (St Quentin-Fallavier, FR). TNF-α was obtained from Invitrogen. This TNF was used in the screen of new necroptosis inhibitors. The chemical synthesis of 6E11 compound is described in Hauteville *et al*.^21^.

### Cell-based screening assay for characterization of necroptosis inhibitors

The cell-based assay used in this study has been previously described by Miao and Degterev^5^. The protocol describing a screening campaign is comprehensively reported in Le Cann *et al*.^22^. Briefly, necroptosis was induced in Jurkat FADD-deficient I 2.1 cell line by addition of 10 ng/ml of human recombinant TNF-α (Invitrogen). Necrostatin-1s (Nec-1s, 7-Cl-O-Nec-1, Calbiochem) was used as positive necroptosis inhibitor. Chemical compounds collection plates were formatted at 10 mM in 100% DMSO in 96-well plates. For each collection plate, two 96-well clear, flat bottom plates (CytoOne, Starlab) were seeded (at 20,000 cells/well) before treatment. One plate was treated with the tested compounds and TNF-α and the other plate was only treated with the tested compounds. Final concentration of each chemical compound was 10 μM at 0,1% DMSO. Cells were incubated at 37 °C, 5% CO_2_ for 24 h before performing MTS viability assay according to the manufacturer’s instructions (CellTiter 96^®^ AQueous Non-Radioactive Cell Proliferation Assay, Promega, Fitchburg, WI, USA). Compounds dilution and distribution in the plates were performed using the Nimbus Microlab liquid handler (Hamilton Robotics, Bonaduz, Switzerland) under microbiological safety workbench.

### Cell death assays

Cell viability was assessed by MTS assay, described here before. Cells were seeded at a density of 2.5 × 10^5^ per well for human PBL or 8 × 10^3^ cells per well for human RPE-1 hTERT cells in 96-well plates and then treated for 24 h. After the treatment, 20 μl of assay solution was added per well for 3 h incubation at 37 °C. The absorbance was determined by a microplate reader (SPECTROstar Nano, BMG Labtech) at 490 nm and 630 nm. The percentage of viability were determined by subtracting the background reading (after 2 h incubation with blank medium) from the absorbance. Results were expressed through the following formula: % viability = (mean (OD_490_ − OD_630_) sample/mean (OD_490_ − OD_630_) control )× 100. Flow cytometry analyses were performed to detect necrotic cells using propidium iodide staining (500 ng/ml) from treated cell lines seeded at a density of 2.5 × 10^5^ cells/well in 24-well plate.

### Cell treatments

Necroptosis was induced by TNF-α or TRAIL treatment in different cellular models. FADD-deficient Jurkat cells were treated with 10 ng/ml human TNF-α for 18 h. Jurkat cells were pre-treated with Z-VAD-fmk (30 µM) and cycloheximide (1 µg/ml) for 30 min and then treated with TRAIL-Flag (200 ng/ml) and 2 µg /ml M2 anti-Flag antibody for 18 h. Apoptosis was induced in wild-type Jurkat cells by treatment with TRAIL-Flag (20 ng/ml) and 2 µg/ml M2 anti-Flag antibody for 18 h. The percentage of propidium iodide positive cells was measured by flow cytometry analysis of fluorescence (FL-2) (FACScalibur, Becton Dickinson, Le Pont de Claix, FR) and expressed as %/NT.

### Cellular ATP concentration measurement

FADD-deficient Jurkat cells were seeded for 24 h at a density of 2 × 10^4^ cells/well in white 96-well plate and then treated with human TNF-α (10 ng/ml). ATP concentration was measured using CellTiter-Glo® Luminescent Cell viability assay kit (Promega) according to the manufacturer’s instruction. ATP concentration was expressed as %/NT ± SD.

### RIPK1 and RIPK3 kinase assays

Human RIPK1 full length GST-tagged was purchased from SignalChem (Richmond, CA, USA). The protocol used to detect the enzymatic activity was adapted from Degterev *et al*.^3^. RIPK1 autophosphorylation was assessed by mixing 5 μl of RIPK1, 5 μl of 3X kinase reaction buffer (5 mM MOPS pH 7.2, 2.5 mM β-glycerophosphate, 4 mM MgCl_2_, 2.5 mM MnCl_2_, 1 mM EGTA, 0.4 mM EDTA, 50 μg/ml BSA, 0.05 mM DTT), 2 μl H_2_O and 3 μl of the tested molecule. This “RIPK1 reaction mix” was incubated on ice for 10 min. During this time, the ATP solution was prepared by mixing 5 μl of 3X kinase reaction buffer, 4 μl H_2_O, 6 μl cold ATP at 150 μM and 2 μCi of [γ-^32^P] ATP. This ATP solution and the RIPK1 reaction mix were then mixed and incubated for 30 min at 30 °C. 25 μl of each reaction were loaded in pre-cast NuPage 12% Bis-Tris gel (Thermo Fisher Scientific) and analyzed by SDS-PAGE. Autophosphorylated RIPK1 band was visualized by analysis in a Typhoon Phosphorimager (GE Healthcare Life Sciences, Velizy-Villacoublay, France). Alternatively, the kinase activity of RIPK1 can be detected using “P81 phosphocellulose assay”. Briefly, 2 µl of kinase was assayed in buffer described above with 0.1 µg/µl of myelin basic protein (MBP, Sigma, #M1891) as substrate and in the presence of 15 µM [γ-^33^P] ATP (3,000 Ci/mmol; 10 mCi/ml). After 30 min incubation at 30 °C, the reaction mix was spotted onto 81mm × 115mm sheets of P81 ion exchange cellulose chromatography paper (Product #IEP-01, Reaction Biology Corp., Malvern, PA, USA) using a FilterMate Harvester (PerkinElmer, Waltham, MA, USA) to remove residual [γ-^33^P] ATP. After extensive washes with a 1% solution of phosphoric acid using a FilterMate Harvester (PerkinElmer, Waltham, MA, USA), the wet filters were counted in the presence of 20 µl ACS (GE Healthcare) scintillation fluid using a TopCount^®^ Scintillation and Luminescence Counter (PerkinElmer, Waltham, MA, USA). Maximal kinase activity was assessed with appropriate dilutions of DMSO. The kinase activity of RIPK3 (human, recombinant, expressed by baculovirus in Sf9 insect cells) was detected with 0.1 µg/µl of MBP as substrate following the assay described above.

### Kinase binding assays

Competition binding assays were performed by DiscoverX (Freemont, CA, USA). Details about these assays can be found in Fabian *et al*.^23^ and Karaman *et al*.^24^ and on the Discoverx website [http://www.discoverx.com/services/drug-discovery-development-services/kinase-profiling/kinomescan]. In this study, binding assays were used for (i) the analysis of selectivity against a large panel of kinases (service “KINOMEscan®”) and for (ii) the determination of binding constant (Kd) of small chemical compound for RIPK1 (service “KdELECT”).

### Mitochondrial transmembrane potential (MTP) assay

Mitochondrial transmembrane potential (MTP) was measured using 3,3′-dihexyloxacarbocyanine iodide, DiOC6(3), and flow cytometry analysis. Human FADD-deficient Jurkat cells were incubated with 6E11 at various concentrations and TNF-α (10 ng/ml) for 18 h. Then treated cells were incubated with DiOC6(3) at 50 nM (the final concentration) for 20 min and investigated for the reduction of MTP by using flow cytometry.

### Molecular docking

According to our previous work relative to RIPK1 ligands^22^, we used a RIP1 kinase homology model including its activation loop to perform docking simulations in the aim to explore the association modes of 6E11 compound to RIPK1. Docking calculations were performed by using VINA standard protocol (YASARA, global docking, 1000 runs theoretical complexes computed). Clustering was achieved through Jarvis-Patrick method as implemented in the Gromacs suite. The best pose of the ligand towards the receptor (energy criteria) was submitted to a 60 ns molecular dynamics (MD) simulation in water to improve the model of 6E11-RIPK1 complex. As the ligand was stable during the MD trajectory, the final model was selected as the energy minimized most representative structure of the simulation (molecular frame at t = 37.570 ns). 6E11-contacting residues were defined as surrounding amino acids given a 3.0 Å cut-off distance and pharmacophores were derived from a standard analysis by using the Ligand Scout program.

### *In vitro* “cold hypoxia/reoxygenation” assays

Human aortic endothelial cells (HAEC; Gibco, Waltham, MA, USA) were grown to 80% confluence, and then synchronized using FBS depleted media for 16 h. For hypothermia/hypoxia, cells were washed twice with PBS then incubated in University of Wisconsin (UW) solution in 95%N_2_/5%CO_2_ atmosphere at 4 °C for 24 h. Increasing concentrations of 6E11, 8A03, Nec-1 and Nec-1s were added in cell medium at various steps: hypoxia; hypoxia and reoxygenation; and only during reoxygenation. Controls are cells not subjected to this protocol and are continuously oxygenated for the same time in PBS supplemented with 2% of FBS. Cell viability was evaluated by XTT test (Sigma-Aldrich, St Quentin-Fallavier, France).

### Statistical analyses

Data from a minimum of two experiments were expressed as means ± range; ± SD or ± SEM. Statistical analyses were done by ANOVA, Tukey’s Multiple Comparison Test and Student’s *t*-test for two groups of data, and significance levels used are *P < 0.05, **P < 0.01, ***P < 0.001 by using GraphPad Prism6 software (GraphPad Software, San Diego, CA, USA).

## Electronic supplementary material

Supplementary tables and figures
