# Supplementary material for: 6E11, a highly selective inhibitor of Receptor-Interacting Protein Kinase 1, protects cells against cold hypoxia-reoxygenation injury
Source: Sci Rep. 2017 Oct 10;7:12931. doi: 10.1038/s41598-017-12788-4 (PMC5635128; doi:10.1038/s41598-017-12788-4)
Supplement: Supplementary file 1 — Supplementary tables and figures [file 41598_2017_12788_MOESM1_ESM.pdf]

SUPPLEMENTARY MATERIAL

6E11, a highly selective inhibitor of Receptor-Interacting Protein Kinase 1, protects cells against cold hypoxia-reoxygenation injury

C Delehouzé[†,1], S Leverrier-Penna[†,2,3], F Le Cann[†,2,3,4,15], A Comte[5], M Jacquard-Fevai [6,7,8,9,10], O Delalande[11], N Desban[1], B Baratte[1], I Gallais[2,3], F Faurez[2,3], MC Bonnet[2,3,12], M Hauteville[13], PG Goekjian[14], R Thuillier[6,7,8,9,10], F Favreau[6,7,8,9,10], P Vandenabeele[4,15], T Hauet[6,7,8,9,10], MT Dimanche-Boitrel[♦,*,2,3] and S Bach[♦,*,1]

[1] Sorbonne Universités, UPMC Univ Paris 06, CNRS USR3151, Protein Phosphorylation and Human Disease Laboratory, Station Biologique, F-29688 Roscoff, France.

[2] INSERM UMR 1085, Institut de Recherche sur la Santé, l'Environnement et le Travail, F-35043 Rennes, France.

[3] Biosit UMS 3080, Université de Rennes 1, F-35043 Rennes, France.

[4] Molecular Signaling and Cell Death Unit, VIB Inflammation Research Center, Ghent, Belgium.

[5] Université de Lyon, CNRS UMR 5246, ICBMS, Chimiothèque, Université Claude Bernard Lyon 1, F-69622 Villeurbanne, France.

[6] Inserm U1082, Poitiers, France.

[7] CHU de Poitiers, Service de Biochimie, Poitiers, France.

[8] Université de Poitiers, Faculté de Médecine et de Pharmacie, Poitiers, France.

[9] Fédération Hospitalo-Universitaire SUPORT, Poitiers, France.

[10] IBiSA Plateforme 'MOPICT', Institut national de la recherche agronomique, Unité expérimentale Génétique, expérimentations et systèmes innovants, Domaine Expérimental du Magneraud, Surgères, France.

[11] CNRS UMR 6290, Institut de Génétique et Développement de Rennes, Université de Rennes 1, F-35043 Rennes, France.

[12] Division of Infection & Immunity, College of Biomedical and Life Sciences, Cardiff University, Cardiff, United Kingdom.

[13] Laboratoire de Biochimie Analytique et Synthèse Bioorganique, Université de Lyon, Université Claude Bernard Lyon 1, F-69622 Villeurbanne, France.

[14] Université de Lyon, CNRS UMR 5246, ICBMS, Laboratoire Chimie Organique 2-Glycosciences, Université Claude Bernard Lyon 1, F-69622 Villeurbanne, France.

[15] Department of Biomedical Molecular Biology, Ghent University, Ghent, Belgium.

[†]These authors contributed equally to this work

[♦] These authors share senior authorship

ref #	References
A	Degterev, A. *et al.*Chemical inhibitor of nonapoptotic cell death with therapeutic potential for ischemic brain injury. *Nat. Chem. Biol.* **1**, 112-119 (2005).
	Northington, F.J., Chavez-Valdez, R. & Martin, L.J. Neuronal cell death in neonatal hypoxia-ischemia. *Ann. Neurol.* **69**, 743-758 (2011).
	Rosenbaum, D.M. *et al.* Necroptosis, a novel form of caspase-independant cell death, contributes to neuronal damage in a retinal ischemia-reperfusion injury model. *J. Neurosci. Res.* **88**, 1569-1576 (2010).
	Stridh, L., Smith, P.L., Naylor, A.S., Wang, X. & Mallard, C. Regultation of toll-like 1 and -2 in neonatal mice brains after hypoxia-ischemia. *J. Neuroinflammation.* **8**, 45; 10.1186/1742-2094-8-45 (2011).
	Oerlemans, M.I. *et al.* Inhibition of RIP1-dependent necrosis prevents adverse cardiac remodeling after myocardial ischemia-reperfusion in vivo. *Basic Res. Cardiol.* **107**, 270; 10.1007/s00395-012-0270-8 (2012).
	Fallach, R. et al. Cardiomyocyte Toll-like receptor 4 is involved in heart dysfunction following septic shock or myocardial ischemia. *J. Mol. Cell Cardiol.* **48**, 1236-1244 (2010).
	Linkermann, A., De Zen, F., Weinberg, J., Kunzendorf, U. & Krautwald, S. Programmed necrosis in acute kidney injury. *Nephrol. Dial. Transplant.* **27**, 3412-3419 (2012).
	Wu, H. *et al.* HMGB1 contributes to kidney ischemia reperfusion injury. *J. Am. Soc. Nephrol.* **21**, 1878-1890 (2010).
	Trichonas, G. *et al.* Receptor interacting protein kinases mediate retinal detachment-induced photoreceptor necrosis and compensate for inhibition of apoptosis. *Proc Natl. Acad. Sci. USA.* **107**, 21695-21700 (2010).
	You, Z. *et al.* Necrostatin-1 reduces histopathology and improves functional outcome after controlled cortical impact in mice. *J. Cereb. Blood Flow Metab.* **28**, 1564-1573 (2008).
	Zhao, H. *et al.* Role of necroptosis in the pathogenesis of solid organ injury. *Cell Death Dis.* **6**, e1975; 10.1038/cddis.2015.316 (2015).
B	Su, Z., Yang, Z., Xie, L., DeWitt, J.P. & Chen, Y. Cancer therapy in the necroptosis era. *Cell Death Differ.* **23**, 748-756 (2016).
	Strilic, B. *et al.* Tumour-cell-induced endothelial cell necroptosis via death receptor 6 promotes metastasis. *Nature.* **536**, 215–218 (2016).
C	Günther, C. et al. Caspase-8 regulates TNF-α-induced epithelial necroptosis and terminal ileitis. *Nature.* **477**, 335-339 (2011).
	Duprez, L. *et al.* RIP kinase-dependent necrosis drives lethal systemic inflammatory response syndrome. *Immunity.* **35**, 908-918 (2011).
	Zhang, W.J. *et al.* Periplogenin induces necroptotic cell death through oxidative stress in HaCaT cells and ameliorates skin lesions in the TPA- and IMQ-induced psoriasis-like mouse models. *Biochem. Pharmacol.* **105**, 66-79 (2016).
	Wang, G., Qu, F.Z., Li, L., Lv, J.C. & Sun, B. Necroptosis: a potential, promising target and switch in acute pancreatitis. *Apoptosis.* **21**, 121-129 (2016).
	Kearney, C.J. & Martin, S.J. An inflammatory perspective on necroptosis. *Mol. Cell.* **65**, 965-973 (2017).
	Rickard, J.A. *et al.* RIPK1 Regulates RIPK3-MLKL-Driven Systemic Inflammation and Emergency Hematopoiesis. *Cell.* **157**, 1175-1188 (2014).
D	Lau, A. *et al.* RIPK3-mediated necroptosis promotes donor kidney inflammatory injury and reduces allograft survival. *Am. J. Transplant.* **13**, 2805-2818 (2013).
	Mannon, R.B. Necroptosis in solid organ transplantation: a missing link to immune activation? *Am. J. Transplant.* **13**, 2785-2786 (2013).
E	Mack, C., Sickmann, A., Lembo, D. & Brune, W. Inhibition of proinflammatory and innate immune signaling pathways by a cytomegalovirus RIP1-interacting protein. *Proc. Natl. Acad. Sci. USA.* **105**, 3094-3099 (2008).
	Jorgensen, I., Rayamajhi, M. & Miao, E.A. Programmed cell death as a defence against infection. *Nat. Rev. Immunol.* **17**, 151-164 (2017).
	Hong, J.R. Betanodavirus: Mitochondrial disruption and necrotic cell death. *World J. Virol.* **2**, 1-5 (2013).
	Berger, A.K. & Danthi, P. Reovirus activates a caspase-independent cell death pathway. *Mbio.* **4**, e00178-13; 10.1128/mBio.00178-13 (2013).
F	Yuan, J. & Kroemer, G. Alternative cell death mechanisms in development and beyond. *Genes Dev.* **24**, 2592-2602 (2010).
	Smith, C.C. & Yellon, D.M. Necroptosis, necrostatins and tissue injury. *J. Cell. Mol. Med.* **15**, 1797-1806 (2011).
	Cougnoux, A. *et al.* Necroptosis in Niemann–Pick disease, type C1: a potential therapeutic target. *Cell Death Dis.* **7**, e2147; doi:10.1038/cddis.2016.16 (2016).
	Shao, L., Yu, S., Ji, W., Li, H. & Gao, Y. The Contribution of Necroptosis in Neurodegenerative Diseases. *Neurochem. Res.* 10.1007/s11064-017-2249-1 (2017).
	Ito, Y. *et al.* RIPK1 mediates axonal degeneration by promoting inflammation and necroptosis in ALS. *Science.* **353**, 603-608 (2016).
G	Jouan-Lanhouet, S. *et al.* TRAIL induces necroptosis involving RIPK1/RIPK3-dependent PARP-1 activation. *Cell Death Differ.* **19**, 2003-2014 (2012).
	Roychowdhury, S., McMullen, M. R., Pisano, S. G., Liu, X. & Nagy, L. E. Absence of receptor interacting protein kinase 3 prevents ethanol-induced liver injury. *Hepatology.* **57**, 1773–1783 (2013).
	Gautheron, J. *et al.* A positive feedback loop between RIP3 and JNK controls non-alcoholic steatohepatitis. *EMBO Mol. Med.* **6**, 1062–1074 (2014).
	Li, J-X. *et al.*The B-RafV600E inhibitor dabrafenib selectively inhibits RIP3 and alleviates acetaminophen-induced liver injury. *Cell Death Dis.* **5**, e1278; 10.1038/cddis.2014.241 (2014).
	Afonso, M. B. *et al.* Necroptosis is a key pathogenic event in human and experimental murine models of non-alcoholic steatohepatitis. *Clin. Sci.* **129**, 721–739 (2015).
	Günther, C. *et al.* The pseudokinase MLKL mediates programmed hepatocellular necrosis independently of RIPK3 during hepatitis. *J. Clin. Invest.* **126**, 4346–4360 (2016).
	Filliol, A. *et al.* RIPK1 protects from TNF-α-mediated liver damage during hepatitis. *Cell Death Dis.* **7**, e2462; 10.1038/cddis.2016.362 (2016).
	Vucur, M. *et al.* RIP3 inhibits inflammatory hepatocarcinogenesis but promotes cholestasis by controlling caspase-8- and JNK-dependent compensatory cell proliferation. *Cell Rep.* **4**, 776-790 (2013).
H	Hanus, J., Andernson, C., Sarraf, D., Ma, J. & Wang, S. Retinal pigment epithelial cell necroptosis in response to sodium iodate. *Cell Death Discov.* **2**, 16054; 10.1038/cddiscovery.2016.54 (2016).
	Hanus, J., Anderson, C. & Wang, S. RPE necroptosis in response to oxidative stress and in AMD. *Ageing Res. Rev.* **24**, 286-298 (2015).

Supplementary Table S1: List of selected articles reporting a link between necroptosis and human diseases (related to Fig. 1).

KINOMESCAN (456 kinases) on 6E11		
**DiscoveRx Gene Symbol**	Entrez Gene Symbol	Percent Control
**AAK1**	AAK1	66
**ABL1-nonphosphorylated**	ABL1	74
**ABL1-phosphorylated**	ABL1	66
**ABL1(E255K)-phosphorylated**	ABL1	86
**ABL1(F317I)-nonphosphorylated**	ABL1	74
**ABL1(F317I)-phosphorylated**	ABL1	91
**ABL1(F317L)-nonphosphorylated**	ABL1	78
**ABL1(F317L)-phosphorylated**	ABL1	84
**ABL1(H396P)-nonphosphorylated**	ABL1	60
**ABL1(H396P)-phosphorylated**	ABL1	83
**ABL1(M351T)-phosphorylated**	ABL1	75
**ABL1(Q252H)-nonphosphorylated**	ABL1	64
**ABL1(Q252H)-phosphorylated**	ABL1	77
**ABL1(T315I)-nonphosphorylated**	ABL1	78
**ABL1(T315I)-phosphorylated**	ABL1	76
**ABL1(Y253F)-phosphorylated**	ABL1	79
**ABL2**	ABL2	100
**ACVR1**	ACVR1	83
**ACVR1B**	ACVR1B	100
**ACVR2A**	ACVR2A	73
**ACVR2B**	ACVR2B	67
**ACVRL1**	ACVRL1	92
**ADCK3**	CABC1	98
**ADCK4**	ADCK4	80
**AKT1**	AKT1	95
**AKT2**	AKT2	84
**AKT3**	AKT3	93
**ALK**	ALK	94
**ALK(C1156Y)**	ALK	71
**ALK(L1196M)**	ALK	64
**AMPK-alpha1**	PRKAA1	80
**AMPK-alpha2**	PRKAA2	98
**ANKK1**	ANKK1	45
**ARK5**	NUAK1	86
**ASK1**	MAP3K5	100

**ASK2**	MAP3K6	86
**AURKA**	AURKA	73
**AURKB**	AURKB	85
**AURKC**	AURKC	94
**AXL**	AXL	89
**BIKE**	BMP2K	94
**BLK**	BLK	85
**BMPR1A**	BMPR1A	100
**BMPR1B**	BMPR1B	91
**BMPR2**	BMPR2	99
**BMX**	BMX	92
**BRAF**	BRAF	87
**BRAF(V600E)**	BRAF	87
**BRK**	PTK6	98
**BRSK1**	BRSK1	100
**BRSK2**	BRSK2	95
**BTK**	BTK	93
**BUB1**	BUB1	71
**CAMK1**	CAMK1	89
**CAMK1D**	CAMK1D	100
**CAMK1G**	CAMK1G	72
**CAMK2A**	CAMK2A	90
**CAMK2B**	CAMK2B	91
**CAMK2D**	CAMK2D	80
**CAMK2G**	CAMK2G	72
**CAMK4**	CAMK4	86
**CAMKK1**	CAMKK1	74
**CAMKK2**	CAMKK2	74
**CASK**	CASK	77
**CDC2L1**	CDK11B	86
**CDC2L2**	CDC2L2	87
**CDC2L5**	CDK13	87
**CDK11**	CDK19	100
**CDK2**	CDK2	100
**CDK3**	CDK3	100
**CDK4-cyclinD1**	CDK4	100
**CDK4-cyclinD3**	CDK4	85
**CDK5**	CDK5	100
**CDK7**	CDK7	73
**CDK8**	CDK8	100
**CDK9**	CDK9	92
**CDKL1**	CDKL1	66
**CDKL2**	CDKL2	95

**CDKL3**	CDKL3	100
**CDKL5**	CDKL5	71
**CHEK1**	CHEK1	96
**CHEK2**	CHEK2	94
**CIT**	CIT	94
**CLK1**	CLK1	91
**CLK2**	CLK2	83
**CLK3**	CLK3	100
**CLK4**	CLK4	98
**CSF1R**	CSF1R	61
**CSF1R-autoinhibited**	CSF1R	55
**CSK**	CSK	89
**CSNK1A1**	CSNK1A1	71
**CSNK1A1L**	CSNK1A1L	92
**CSNK1D**	CSNK1D	100
**CSNK1E**	CSNK1E	65
**CSNK1G1**	CSNK1G1	84
**CSNK1G2**	CSNK1G2	100
**CSNK1G3**	CSNK1G3	100
**CSNK2A1**	CSNK2A1	79
**CSNK2A2**	CSNK2A2	84
**CTK**	MATK	53
**DAPK1**	DAPK1	100
**DAPK2**	DAPK2	80
**DAPK3**	DAPK3	81
**DCAMKL1**	DCLK1	71
**DCAMKL2**	DCLK2	90
**DCAMKL3**	DCLK3	88
**DDR1**	DDR1	99
**DDR2**	DDR2	68
**DLK**	MAP3K12	93
**DMPK**	DMPK	96
**DMPK2**	CDC42BPG	100
**DRAK1**	STK17A	82
**DRAK2**	STK17B	80
**DYRK1A**	DYRK1A	74
**DYRK1B**	DYRK1B	70
**DYRK2**	DYRK2	71
**EGFR**	EGFR	100
**EGFR(E746-A750del)**	EGFR	100
**EGFR(G719C)**	EGFR	94
**EGFR(G719S)**	EGFR	100
**EGFR(L747-E749del, A750P)**	EGFR	100

**EGFR(L747-S752del, P753S)**	EGFR	100
**EGFR(L747-T751del,Sins)**	EGFR	97
**EGFR(L858R,T790M)**	EGFR	100
**EGFR(L858R)**	EGFR	100
**EGFR(L861Q)**	EGFR	92
**EGFR(S752-I759del)**	EGFR	85
**EGFR(T790M)**	EGFR	100
**EIF2AK1**	EIF2AK1	59
**EPHA1**	EPHA1	100
**EPHA2**	EPHA2	92
**EPHA3**	EPHA3	100
**EPHA4**	EPHA4	93
**EPHA5**	EPHA5	100
**EPHA6**	EPHA6	96
**EPHA7**	EPHA7	100
**EPHA8**	EPHA8	100
**EPHB1**	EPHB1	100
**EPHB2**	EPHB2	100
**EPHB3**	EPHB3	95
**EPHB4**	EPHB4	96
**EPHB6**	EPHB6	79
**ERBB2**	ERBB2	85
**ERBB3**	ERBB3	90
**ERBB4**	ERBB4	100
**ERK1**	MAPK3	100
**ERK2**	MAPK1	100
**ERK3**	MAPK6	100
**ERK4**	MAPK4	92
**ERK5**	MAPK7	77
**ERK8**	MAPK15	94
**ERN1**	ERN1	58
**FAK**	PTK2	100
**FER**	FER	88
**FES**	FES	95
**FGFR1**	FGFR1	99
**FGFR2**	FGFR2	100
**FGFR3**	FGFR3	98
**FGFR3(G697C)**	FGFR3	100
**FGFR4**	FGFR4	98
**FGR**	FGR	92
**FLT1**	FLT1	100
**FLT3**	FLT3	63
**FLT3-autoinhibited**	FLT3	87

**FLT3(D835H)**	FLT3	100
**FLT3(D835Y)**	FLT3	100
**FLT3(ITD)**	FLT3	98
**FLT3(K663Q)**	FLT3	82
**FLT3(N841I)**	FLT3	86
**FLT3(R834Q)**	FLT3	89
**FLT4**	FLT4	100
**FRK**	FRK	100
**FYN**	FYN	100
**GAK**	GAK	86
**GCN2(Kin.Dom.2,S808G)**	EIF2AK4	82
**GRK1**	GRK1	84
**GRK4**	GRK4	100
**GRK7**	GRK7	81
**GSK3A**	GSK3A	85
**GSK3B**	GSK3B	82
**HASPIN**	GSG2	97
**HCK**	HCK	61
**HIPK1**	HIPK1	73
**HIPK2**	HIPK2	100
**HIPK3**	HIPK3	77
**HIPK4**	HIPK4	100
**HPK1**	MAP4K1	93
**HUNK**	HUNK	70
**ICK**	ICK	90
**IGF1R**	IGF1R	100
**IKK-alpha**	CHUK	75
**IKK-beta**	IKBKB	66
**IKK-epsilon**	IKBKE	69
**INSR**	INSR	84
**INSRR**	INSRR	100
**IRAK1**	IRAK1	75
**IRAK3**	IRAK3	100
**IRAK4**	IRAK4	91
**ITK**	ITK	97
**JAK1(JH1domain-catalytic)**	JAK1	82
**JAK1(JH2domain-pseudokinase)**	JAK1	90
**JAK2(JH1domain-catalytic)**	JAK2	97
**JAK3(JH1domain-catalytic)**	JAK3	75
**JNK1**	MAPK8	68
**JNK2**	MAPK9	57
**JNK3**	MAPK10	67
**KIT**	KIT	88

**KIT-autoinhibited**	KIT	74
**KIT(A829P)**	KIT	98
**KIT(D816H)**	KIT	100
**KIT(D816V)**	KIT	98
**KIT(L576P)**	KIT	92
**KIT(V559D,T670I)**	KIT	91
**KIT(V559D,V654A)**	KIT	76
**KIT(V559D)**	KIT	61
**LATS1**	LATS1	88
**LATS2**	LATS2	64
**LCK**	LCK	86
**LIMK1**	LIMK1	69
**LIMK2**	LIMK2	100
**LKB1**	STK11	100
**LOK**	STK10	93
**LRRK2**	LRRK2	93
**LRRK2(G2019S)**	LRRK2	100
**LTK**	LTK	80
**LYN**	LYN	100
**LZK**	MAP3K13	100
**MAK**	MAK	99
**MAP3K1**	MAP3K1	77
**MAP3K15**	MAP3K15	67
**MAP3K2**	MAP3K2	89
**MAP3K3**	MAP3K3	70
**MAP3K4**	MAP3K4	100
**MAP4K2**	MAP4K2	66
**MAP4K3**	MAP4K3	100
**MAP4K4**	MAP4K4	66
**MAP4K5**	MAP4K5	72
**MAPKAPK2**	MAPKAPK2	100
**MAPKAPK5**	MAPKAPK5	88
**MARK1**	MARK1	90
**MARK2**	MARK2	93
**MARK3**	MARK3	100
**MARK4**	MARK4	88
**MAST1**	MAST1	78
**MEK1**	MAP2K1	89
**MEK2**	MAP2K2	84
**MEK3**	MAP2K3	73
**MEK4**	MAP2K4	86
**MEK5**	MAP2K5	35
**MEK6**	MAP2K6	85

**MELK**	MELK	98
**MERTK**	MERTK	100
**MET**	MET	100
**MET(M1250T)**	MET	91
**MET(Y1235D)**	MET	98
**MINK**	MINK1	51
**MKK7**	MAP2K7	80
**MKNK1**	MKNK1	66
**MKNK2**	MKNK2	68
**MLCK**	MYLK3	88
**MLK1**	MAP3K9	100
**MLK2**	MAP3K10	100
**MLK3**	MAP3K11	92
**MRCKA**	CDC42BPA	84
**MRCKB**	CDC42BPB	100
**MST1**	STK4	92
**MST1R**	MST1R	100
**MST2**	STK3	86
**MST3**	STK24	100
**MST4**	MST4	65
**MTOR**	MTOR	70
**MUSK**	MUSK	100
**MYLK**	MYLK	67
**MYLK2**	MYLK2	100
**MYLK4**	MYLK4	99
**MYO3A**	MYO3A	91
**MYO3B**	MYO3B	83
**NDR1**	STK38	62
**NDR2**	STK38L	88
**NEK1**	NEK1	89
**NEK10**	NEK10	67
**NEK11**	NEK11	89
**NEK2**	NEK2	85
**NEK3**	NEK3	75
**NEK4**	NEK4	64
**NEK5**	NEK5	100
**NEK6**	NEK6	100
**NEK7**	NEK7	89
**NEK9**	NEK9	83
**NIK**	MAP3K14	54
**NIM1**	MGC42105	83
**NLK**	NLK	100
**OSR1**	OXSR1	87

**p38-alpha**	MAPK14	52
**p38-beta**	MAPK11	98
**p38-delta**	MAPK13	100
**p38-gamma**	MAPK12	59
**PAK1**	PAK1	100
**PAK2**	PAK2	90
**PAK3**	PAK3	90
**PAK4**	PAK4	100
**PAK6**	PAK6	89
**PAK7**	PAK7	93
**PCTK1**	CDK16	75
**PCTK2**	CDK17	77
**PCTK3**	CDK18	89
**PDGFRA**	PDGFRA	79
**PDGFRB**	PDGFRB	98
**PDPK1**	PDPK1	100
**PfCDPK1 (*P. falciparum*)**	CDPK1	60
**PfPK5 (*P. falciparum*)**	MAL13P1.279	70
**PFTAIRE2**	CDK15	72
**PFTK1**	CDK14	85
**PHKG1**	PHKG1	100
**PHKG2**	PHKG2	69
**PIK3C2B**	PIK3C2B	80
**PIK3C2G**	PIK3C2G	69
**PIK3CA**	PIK3CA	79
**PIK3CA(C420R)**	PIK3CA	72
**PIK3CA(E542K)**	PIK3CA	65
**PIK3CA(E545A)**	PIK3CA	80
**PIK3CA(E545K)**	PIK3CA	63
**PIK3CA(H1047L)**	PIK3CA	53
**PIK3CA(H1047Y)**	PIK3CA	96
**PIK3CA(I800L)**	PIK3CA	70
**PIK3CA(M1043I)**	PIK3CA	71
**PIK3CA(Q546K)**	PIK3CA	64
**PIK3CB**	PIK3CB	62
**PIK3CD**	PIK3CD	63
**PIK3CG**	PIK3CG	68
**PIK4CB**	PI4KB	87
**PIM1**	PIM1	100
**PIM2**	PIM2	100
**PIM3**	PIM3	100
**PIP5K1A**	PIP5K1A	100
**PIP5K1C**	PIP5K1C	78

**PIP5K2B**	PIP4K2B	100
**PIP5K2C**	PIP4K2C	71
**PKAC-alpha**	PRKACA	100
**PKAC-beta**	PRKACB	93
**PKMYT1**	PKMYT1	100
**PKN1**	PKN1	100
**PKN2**	PKN2	99
**PKNB (*M. tuberculosis*)**	pknB	92
**PLK1**	PLK1	81
**PLK2**	PLK2	67
**PLK3**	PLK3	79
**PLK4**	PLK4	78
**PRKCD**	PRKCD	91
**PRKCE**	PRKCE	77
**PRKCH**	PRKCH	100
**PRKCI**	PRKCI	60
**PRKCQ**	PRKCQ	93
**PRKD1**	PRKD1	77
**PRKD2**	PRKD2	90
**PRKD3**	PRKD3	100
**PRKG1**	PRKG1	89
**PRKG2**	PRKG2	71
**PRKR**	EIF2AK2	77
**PRKX**	PRKX	100
**PRP4**	PRPF4B	100
**PYK2**	PTK2B	90
**QSK**	KIAA0999	62
**RAF1**	RAF1	100
**RET**	RET	68
**RET(M918T)**	RET	100
**RET(V804L)**	RET	100
**RET(V804M)**	RET	100
**RIOK1**	RIOK1	100
**RIOK2**	RIOK2	76
**RIOK3**	RIOK3	100
**RIPK1**	RIPK1	0.15
**RIPK2**	RIPK2	100
**RIPK4**	RIPK4	83
**RIPK5**	DSTYK	90
**ROCK1**	ROCK1	89
**ROCK2**	ROCK2	100
**ROS1**	ROS1	87
**RPS6KA4(Kin.Dom.1-N-terminal)**	RPS6KA4	96

**RPS6KA4(Kin.Dom.2-C-terminal)**	RPS6KA4	81
**RPS6KA5(Kin.Dom.1-N-terminal)**	RPS6KA5	100
**RPS6KA5(Kin.Dom.2-C-terminal)**	RPS6KA5	92
**RSK1(Kin.Dom.1-N-terminal)**	RPS6KA1	92
**RSK1(Kin.Dom.2-C-terminal)**	RPS6KA1	100
**RSK2(Kin.Dom.1-N-terminal)**	RPS6KA3	83
**RSK2(Kin.Dom.2-C-terminal)**	RPS6KA3	76
**RSK3(Kin.Dom.1-N-terminal)**	RPS6KA2	100
**RSK3(Kin.Dom.2-C-terminal)**	RPS6KA2	96
**RSK4(Kin.Dom.1-N-terminal)**	RPS6KA6	87
**RSK4(Kin.Dom.2-C-terminal)**	RPS6KA6	100
**S6K1**	RPS6KB1	63
**SBK1**	SBK1	78
**SGK**	SGK1	82
**SgK110**	SgK110	85
**SGK2**	SGK2	57
**SGK3**	SGK3	56
**SIK**	SIK1	100
**SIK2**	SIK2	100
**SLK**	SLK	99
**SNARK**	NUAK2	55
**SNRK**	SNRK	54
**SRC**	SRC	97
**SRMS**	SRMS	66
**SRPK1**	SRPK1	100
**SRPK2**	SRPK2	100
**SRPK3**	SRPK3	100
**STK16**	STK16	89
**STK33**	STK33	100
**STK35**	STK35	100
**STK36**	STK36	100
**STK39**	STK39	81
**SYK**	SYK	85
**TAK1**	MAP3K7	77
**TAOK1**	TAOK1	64
**TAOK2**	TAOK2	66
**TAOK3**	TAOK3	74
**TBK1**	TBK1	85
**TEC**	TEC	98
**TESK1**	TESK1	100
**TGFBR1**	TGFBR1	100
**TGFBR2**	TGFBR2	95
**TIE1**	TIE1	100

**TIE2**	TEK	100
**TLK1**	TLK1	100
**TLK2**	TLK2	83
**TNIK**	TNIK	85
**TNK1**	TNK1	88
**TNK2**	TNK2	92
**TNNI3K**	TNNI3K	98
**TRKA**	NTRK1	93
**TRKB**	NTRK2	100
**TRKC**	NTRK3	100
**TRPM6**	TRPM6	60
**TSSK1B**	TSSK1B	83
**TTK**	TTK	99
**TXK**	TXK	100
**TYK2(JH1domain-catalytic)**	TYK2	97
**TYK2(JH2domain-pseudokinase)**	TYK2	86
**TYRO3**	TYRO3	93
**ULK1**	ULK1	79
**ULK2**	ULK2	98
**ULK3**	ULK3	71
**VEGFR2**	KDR	82
**VRK2**	VRK2	58
**WEE1**	WEE1	82
**WEE2**	WEE2	88
**WNK1**	WNK1	61
**WNK3**	WNK3	50
**YANK1**	STK32A	60
**YANK2**	STK32B	84
**YANK3**	STK32C	72
**YES**	YES1	87
**YSK1**	STK25	88
**YSK4**	YSK4	81
**ZAK**	ZAK	100
**ZAP70**	ZAP70	100

Supplementary Table S2: Matrix of screen for the selectivity profiling of 6E11 (related to Fig. 5a). The table reports the full list of the 456 kinases used in the selectivity profiling of 6E11. 6E11 was assayed at 10 μM and results are reported as "% Ctrl", where lower numbers indicate stronger hits in the panel of kinases tested. %Ctrl calculation: (test compound signal - positive control signal)/(negative control signal - positive control signal) x 100. Test compound = 6E11; negative control = DMSO (100%Ctrl); positive control = control compound (0%Ctrl).

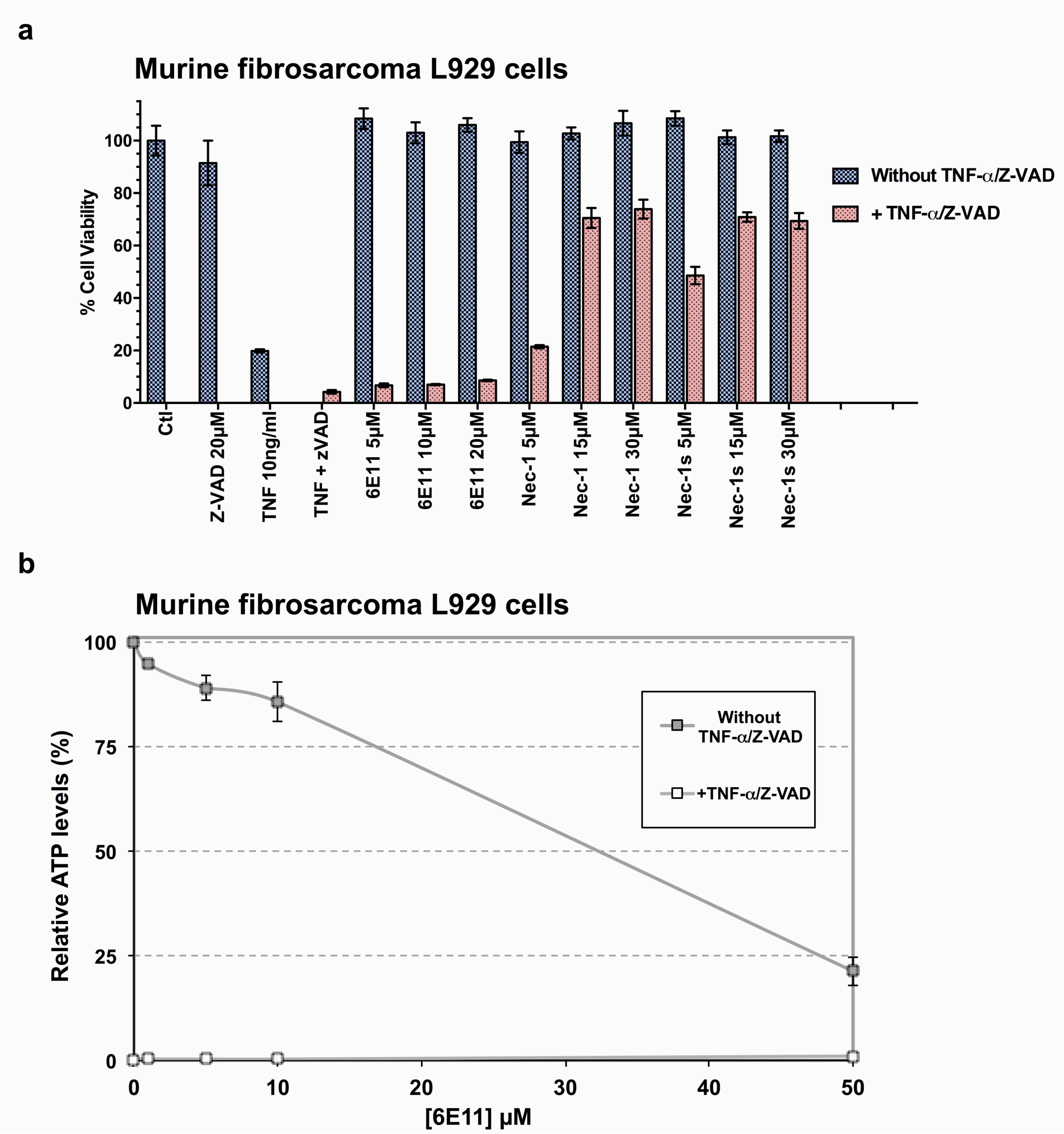

Supplementary Figure S1: **6E11 is inactive against TNF-α-induced necroptosis in L929 murine fibrosarcoma cells.** (a) Dose-dependent effect of 6E11 against TNF-α/Z-VAD induced mouse L929 cell necroptosis. After a 24-h incubation of the cells with or without 10ng/ml of TNF-α and 20 µM of Z-VAD and increasing concentrations of tested compounds (6E11, Nec-1 and Nec-1s), the effect on the cell viability was evaluated by MTS reduction assay. The values were normalized as a percentage of cell viability, considering 100% viable cells in the control treated with DMSO (n=3, mean ± SD). (b) L929 murine fibrosarcoma cells were treated or not with TNF-α (10 ng/ml)/ Z-VAD (20 µM) in presence or not of increasing concentrations of 6E11 (0, 1, 5, 10, 50 µM) for 18 hours. Intracellular ATP levels were measured with the CellTiter-Glo® Luminescent Cell Viability Assay (n=3, mean ± SEM).

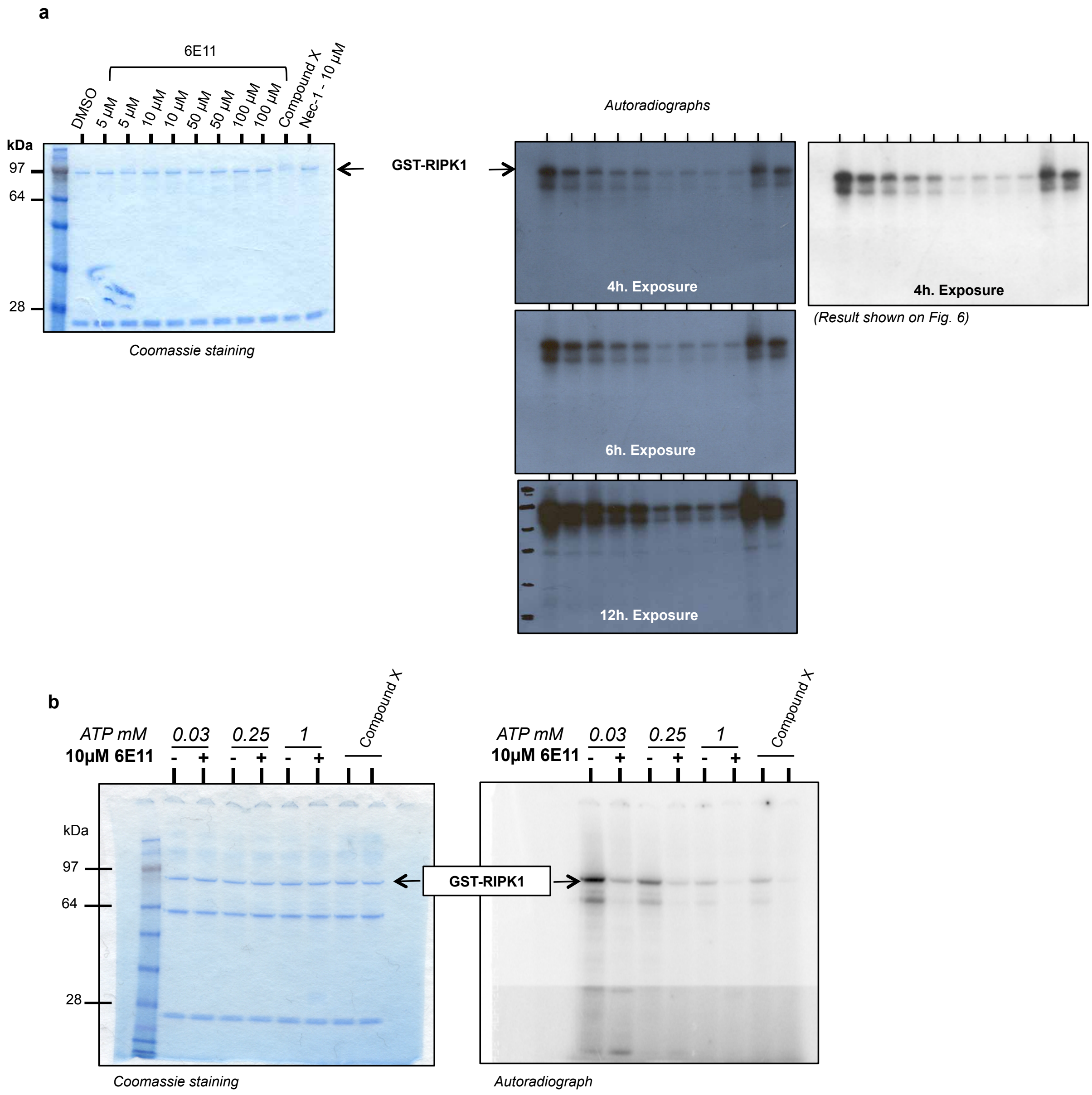

Supplementary Figure S2: **6E11 inhibits the enzymatic activity of RIPK1 with a non-ATP competitive mode of action (related to Fig. 6).** (a) RIPK1 was treated with 5, 10, 50 and 100 µM of 6E11 to analyze the effect on the kinase autophosphorylation. Radioactive autophosphorylation assays were processed with $[\gamma\text{-}^{32}P]$ ATP at 30 µM final concentration. Necrostatin-1 (Nec-1) was used as an internal control. Coomassie blue staining was performed in order to estimate the total amount of protein loaded on polyacrylamide gel. Autophosphorylated RIPK1 band was visualized on radiographic film. Results at various exposure times and full-lengh gel are shown here. Compound X is not described in this article. (b) ATP competition assay shows that inhibition of RIPK1 activity by 6E11 is not affected by ATP concentration. 0.25 or 1 mM of cold ATP were used to study the competition with 6E11. Compound X is not described in this article. These assays are similar to those described in (a). The phosphorylation signal is modified by increasing doses of ATP due to the dilution of $[\gamma\text{-}^{32}P]$ ATP by "cold" non radiolabeled ATP. Note here that two different batches of active RIPK1 were used to perform the experiments shown on panels a and b. This could explain the difference observed after coomassie staining. The additionnal band observed on panel b (coomassie staining) at approximately 64 kDa has no impact on the result and our interpretation as it was not labelled radioactively.

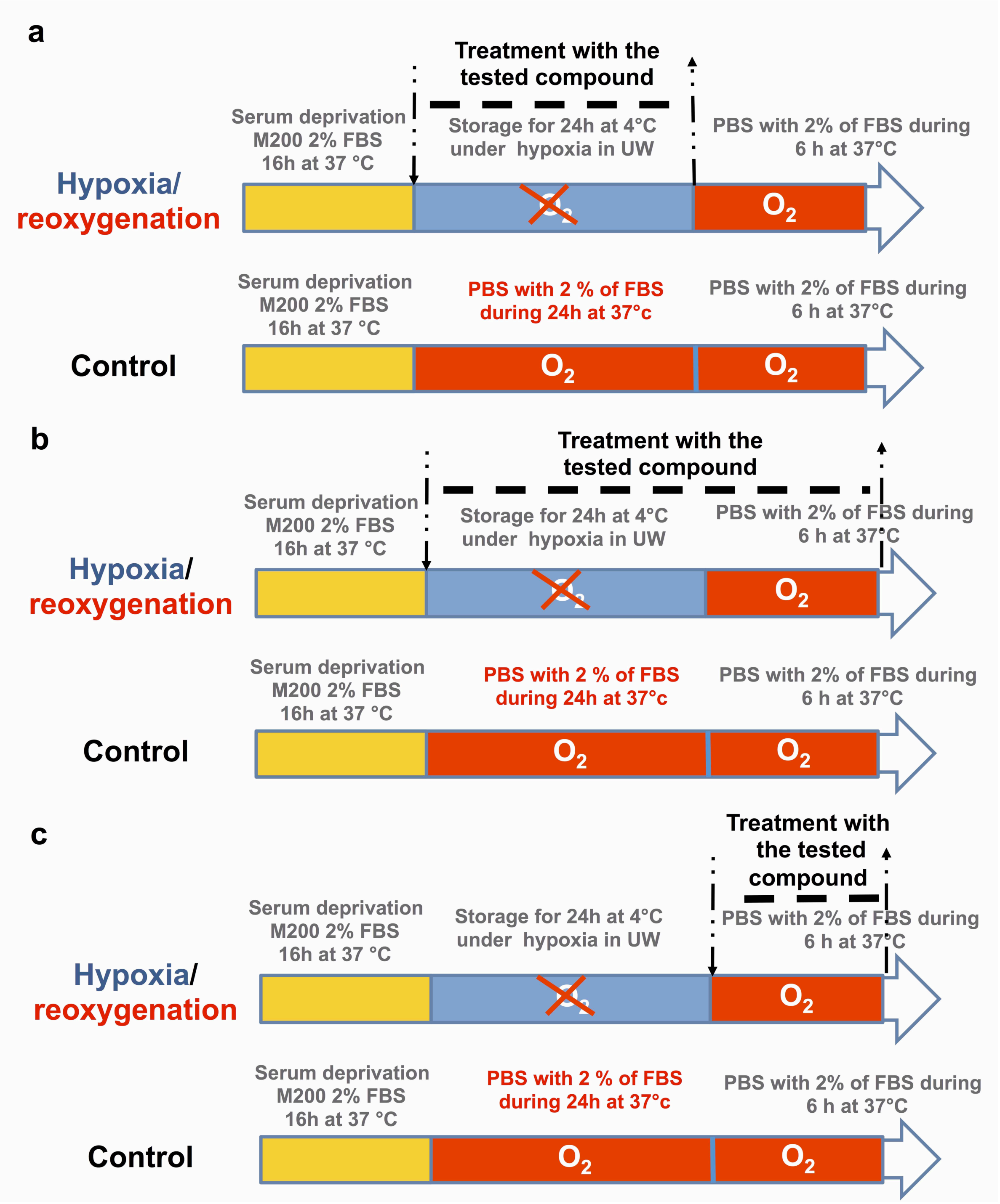

Supplementary Figure S3: Schematic views of the protocols performed to detect the impact of RIPK1 inhibitors on the protection of human aortic endothelial cells (HAEC) subjected to hypoxia reoxygenation injury, when treatment is performed during cold hypoxia step (a), during cold hypoxia and reoxygenation steps (b) and during the reoxygenation step (c) (related to Fig. 8).
